# Missense variants in CMS22 patients reveal that PREPL has both enzymatic and nonenzymatic functions

**DOI:** 10.1172/jci.insight.179276

**Published:** 2024-09-10

**Authors:** Yenthe Monnens, Anastasia Theodoropoulou, Karen Rosier, Kritika Bhalla, Alexia Mahy, Roeland Vanhoutte, Sandra Meulemans, Edoardo Cavani, Aleksandar Antanasijevic, Irma Lemmens, Jennifer A. Lee, Catherine J. Spellicy, Richard J. Schroer, Ricardo A. Maselli, Chamindra G. Laverty, Patrizia Agostinis, David J. Pagliarini, Steven Verhelst, Maria J. Marcaida, Anne Rochtus, Matteo Dal Peraro, John W.M. Creemers

**Affiliations:** 1 Laboratory for Biochemical Neuroendocrinology, Department of Human Genetics, KU Leuven, Leuven, Belgium.; 2 Laboratory for Biomolecular Modeling, Institute of Bioengineering, School of Life Sciences, École Polytechnique Fédérale de Lausanne (EPFL), Lausanne, Switzerland.; 3 Laboratory for Chemical Biology, Department of Cellular and Molecular Medicine, KU Leuven, Leuven, Belgium.; 4 Global Health Institute, School of Life Sciences, École Polytechnique Fédérale de Lausanne (EPFL), Lausanne, Switzerland.; 5 VIB-UGent Center for Medical Biotechnology, Department of Biomolecular Medicine, Faculty of Medicine and Health Sciences, Ghent University, Ghent, Belgium.; 6 Greenwood Genetic Center, South Carolina, USA.; 7 Department of Neurology, UCD, Sacramento, California, USA.; 8 Department of Neuroscience, UCSD, San Diego, California, USA.; 9 Laboratory for Cell death Research & Therapy, VIB, Department of Cellular and Molecular Medicine, KU Leuven, Leuven, Belgium.; 10 Department of Biochemistry, University of Wisconsin-Madison, Madison, Wisconsin, USA.; 11 UZ Leuven University Hospital, Leuven, Belgium.

**Keywords:** Endocrinology, Genetics, Genetic variation

## Abstract

Congenital myasthenic syndrome-22 (CMS22, OMIM 616224) is a rare genetic disorder caused by deleterious genetic variation in the prolyl endopeptidase-like (*PREPL*) gene. Previous reports have described patients with deletions and nonsense variants in *PREPL*, but nothing is known about the effect of missense variants in the pathology of CMS22. In this study, we have functionally characterized missense variants in *PREPL* from 3 patients with CMS22, all with hallmark phenotypes. Biochemical evaluation revealed that these missense variants do not impair hydrolase activity, thereby challenging the conventional diagnostic criteria and disease mechanism. Structural analysis showed that the variants affect regions most likely involved in intraprotein or protein-protein interactions. Indeed, binding to a selected group of known interactors was differentially reduced for the 3 variants. The importance of nonhydrolytic functions of PREPL was investigated in catalytically inactive PREPL p.Ser559Ala cell lines, which showed that hydrolytic activity of PREPL is needed for normal mitochondrial function but not for regulating AP1-mediated transport in the transgolgi network. In conclusion, these studies showed that CMS22 can be caused not only by deletion and truncation of PREPL but also by missense variants that do not necessarily result in a loss of hydrolytic activity of PREPL.

## Abstract 2

We identified that missense mutations in *PREPL* can lead to CMS22 by disrupting protein-protein interactions of PREPL without altering catalytic activity of PREPL.

## Graphical abstract

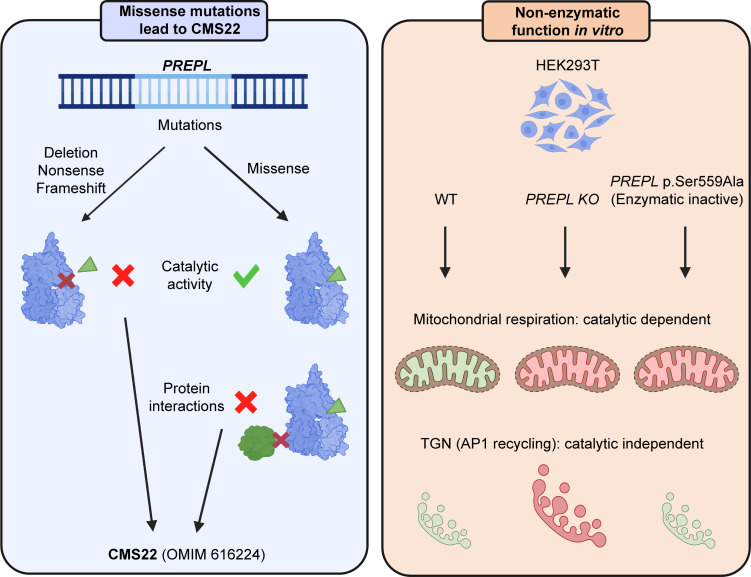

## Introduction

Congenital myasthenic syndrome-22 (CMS22, OMIM 616224) is a rare recessive disorder caused by deleterious genetic and genomic variation in the prolyl endopeptidase-like (*PREPL*) gene (1–6). Patients experience severe neonatal hypotonia, eyelid ptosis, feeding problems, and growth hormone deficiency. During childhood, the hypotonia spontaneously improves and patients develop hyperphagia and rapid weight gain (1, 2, 4, 6–13). Approximately half of the patients experience learning difficulties and have an average IQ of 70 (3). Previous reports have mostly documented patients with CMS22 harboring partial or complete loss of *PREPL* alone or together with flanking genes (1, 2, 4, 6–13). In addition, a small number of point mutations in the *PREPL* gene have been identified, which lead to a frameshift or premature stop codon (1–6). Recently, a patient with a missense variant in *PREPL* was reported but not functionally characterized (14).

The PREPL protein consists of 2 structural domains: an amino-terminal 7-bladed β-propeller domain and a carboxy-terminal α/β-hydrolase fold catalytic domain connected by a hinge loop (8, 15). The active site is comprised of the triad residues Ser559, Asp645, and His690. Two main isoforms of PREPL exist, a short form, PREPL_S_ (683 aa), and a long form, PREPL_L_ (727 aa). PREPL_S_ is localized in the cytosol while PREPL_L_ contains an N-terminal mitochondrial targeting sequence that translocates PREPL into the mitochondria (15–18). Experimental evidence confirms the catalytic activity of PREPL as it reacts with the activity-based probe FP-biotin, which is derived from the serine hydrolase inhibitor diisopropyl fluorophosphates and can be inhibited by serine hydrolase inhibitors including PMSF and AEBSF (2, 6, 7). However, in contrast to its homologues PREP and OpdB, PREPL does not appear to possess protease activity despite the structural similarities (10, 11). Recently, PREPL was found to hydrolyze chromogenic ester and thioester substrates in vitro and its activity was inhibited by Palmostatin M, a known inhibitor for acyl protein thioesterases 1 and 2 (APT1 and APT2) (15). Therefore, a depalmitoylating function has been proposed, as an increase in protein palmitoylation is also evident in PREPL-deficient cells (19). Nevertheless, no physiological PREPL substrates have been identified to date.

In addition to its catalytic function, several lines of evidence support noncatalytic functions for PREPL. Firstly, yeast-two-hybrid experiments identified an interaction between PREPL and the μ1A subunit of the adaptor protein complex AP-1 (20). AP-1 function is to bind to the membrane of the trans golgi network (TGN) and endosomes where it facilitates clathrin-mediated vesicle formation. Notably, PREPL-deficient human skin fibroblasts showed prolonged membrane binding of AP-1 and a TGN expansion, showing that PREPL facilitates AP-1 membrane recycling. This phenotype was rescued with a catalytically inactive PREPL p.Ser559Ala variant, suggesting a catalytic independent mechanism. Moreover, PREPL interacts with α-synuclein, thereby influencing aggregation without α-synuclein cleavage (21). For PREP, a homologue of PREPL, substrate binding in the catalytic site is thought to result in an allosteric change that alters protein-protein interactions (22, 23). It was further hypothesized that these interactions are physiologically more relevant compared with isolated hydrolytic activity, and that substrate or inhibitor binding and cleavage has only a modulatory role to the function of PREP. Indeed, PREP can mediate cellular processes independent of catalytic activity (24–29). A similar mechanism of action could be valid for PREPL as well.

In this study, we describe 2 new patients and 1 published but not functionally characterized patient with CMS22 who harbor missense variants in *PREPL*. We have studied the effect of the variants on catalytic activity, protein stability, and protein-protein interactions. Experimental data suggest that compromising either the catalytic or noncatalytic functions of PREPL can result in CMS22. Using a HEK293T cell line harboring a p.Ser559Ala mutation in PREPL we investigated the need for catalytic activity of PREPL to maintain normal TGN morphology and mitochondrial respiration. Finally, we propose a new model for the physiological function of PREPL that integrates both its catalytic and noncatalytic functions.

## Results

### Three missense variants in PREPL identified in patients with CMS22.

Here we present 2 novel patients with CMS22 with monoallelic missense variants in *PREPL* combined with a monoallelic frameshift and premature stop codon. In addition, a recent publication described a new patient with CMS22 with a homozygous missense variant in *PREPL* (1). An overview of patient characteristics is shown in Table 1.

Patient 1 is biologically female and born by vaginal delivery at 41 weeks and 6 days postinduction. The pregnancy was unremarkable, although the fetus had reduced movements. She presented with severe neonatal hypotonia and feeding difficulties. Nasogastric tube feeding was started at 6 weeks of age. She presented intermittent mild ptosis but otherwise had a normal ophthalmologic examination, a high-arched palate, and no other facial weakness. She was markedly hypotonic, more pronounced in the upper extremities. Muscle tone was better in her distal upper extremities and in her legs. Deep tendon reflexes (DTRs) were decreased but plantar responses were bilaterally in flexion. No electrodiagnostic tests have been performed. She achieved independent sitting at 8 months and independent walking at 18 months. Brain MRI was normal but spine MRI demonstrated a syrinx (2 mm diameter) from T8 to conus without evidence of myelopathy. Laboratory analysis showed a mildly low circulating insulin-like growth factor-I of 16 ng/mL (ref 17; 185 ng/ml) but with a normal insulin-like growth factor binding protein 3 (IGFBP3) for her age, in which the former normalized over time without treatment. Urinary amino acids did not show cystinuria. She is now 4 years old and doing well on pyridostigmine and albuterol oral syrup. Current exam shows mild myopathic facies, high-arched palate, normal extraocular movements and facial expression, nasal speech, normal DTR, normal run, walk, and rise from floor but with fatigue after high levels of activity only. She has steadily gained weight from the 1st percentile at birth to 36th percentile at 4 years of age. She was never treated with growth hormone. Language and cognitive development are normal and she is behaviorally and socially age appropriate. Sequencing identified a paternally inherited c.727C>T variant resulting in p.Arg243Cys and a maternally inherited duplication resulting in a frameshift and early stop codon p.Leu597Hisfs*4 in *PREPL* (NM 006036.4).

Patient 2 is biologically male and born at 39 weeks by caesarean section for failure to progress after an uncomplicated pregnancy. His birth weight was 2,825 g (–1.4 SD), length 47 cm (–1.6 SD), and head circumference 34 cm (–0.5 SD). He had profound neonatal hypotonia, poor sucking, and feeding difficulties, which persisted throughout infancy and childhood. No electrodiagnostic tests have been performed. He had slow weight gain and was underweight for his length in infancy. Starting from 6 years of age he had rapid weight gain. He started sitting and crawling at 12 months of age and walking at 24 months. He received physiotherapy, occupational therapy, and speech therapy. He seemed weaker and more tired later in the day. He also had occasional upper eyelid ptosis later in the day. He was diagnosed with obstructive sleep apnea during a pediatric nocturnal polysomnogram at 21 months of age. He was on CPAP/BiPAP until 3 years of age. Laboratory tests repeatedly showed normal IGF-I and IGFBP3. Urinary amino acids did not show cystinuria. Patient 2 is currently 7 years and 11 months old with weight of 24.7 kg (42nd percentile), height 116 cm (2nd percentile), and head circumference 51.7 cm (32nd percentile). He has nasal speech, normal facial expression, movement, and strength. There is no ptosis. He has normal DTRs but an awkward tandem gait. He is social and learns well at school; there are no behavioral problems. Sequencing identified a paternal c.1235T>G variant resulting in p.Ile412Arg and a maternal c.1978_1981delCTCA variant leading to p.Leu660Argfs*22 in *PREPL* (NM 006036.4).

Patient 3 was recently described by Kim et al. (2020) and was included in our biochemical analysis to characterize the pathogenicity of the missense variant on PREPL functioning. Patient 3 is biologically male and is the first child of nonconsanguineous Korean parents. He was born at 34 gestational weeks with a birth weight of 1,620 g (–4.39 SD), a height of 41 cm (–4.69 SD), and a head circumference of 30.5 cm (SD, −3.12 SD). Caesarean section delivery was performed because of oligohydramnios. Immediately after birth, he was admitted to the neonatal intensive care unit due to prematurity and respiratory distress. On physical examination at birth, dysmorphic features were noted, including thick arched eyebrows, hypertelorism, a broad prominent nasal bridge, low set ears, a cleft and high arched palate, and a short webbed neck. The patient showed global muscle hypotonia with limited spontaneous movement and decreased deep tendon reflex. The patient also had multiple additional anomalies, including hearing disturbance, supravalvular aortic stenosis, a tethered spinal cord, cryptorchidism, and duplex kidney. No electrodiagnostic tests have been performed. The brain magnetic resonance image (MRI) at 1 month of age showed no specific abnormality. Laboratory tests, including a metabolic profile, plasma amino acids, urine organic acids, plasma acylcarnitines, and thyroid function were unremarkable. The karyotyping, multiplex ligation-dependent probe amplification (MLPA) analysis for 26 microdeletion syndromes, and the diagnostic exome sequencing of 4,813 OMIM genes were also normal. At 2 years old, the patient presented global developmental delay, barely sat with support, and only spoke a single word. Whole exome sequencing identified a homozygous c.1940G>A variant resulting in p.Arg647Gln in *PREPL* (NM 006036.4).

### Catalytic activity is conserved in CMS22 patient-derived PREPL variants.

**Figure 1:**
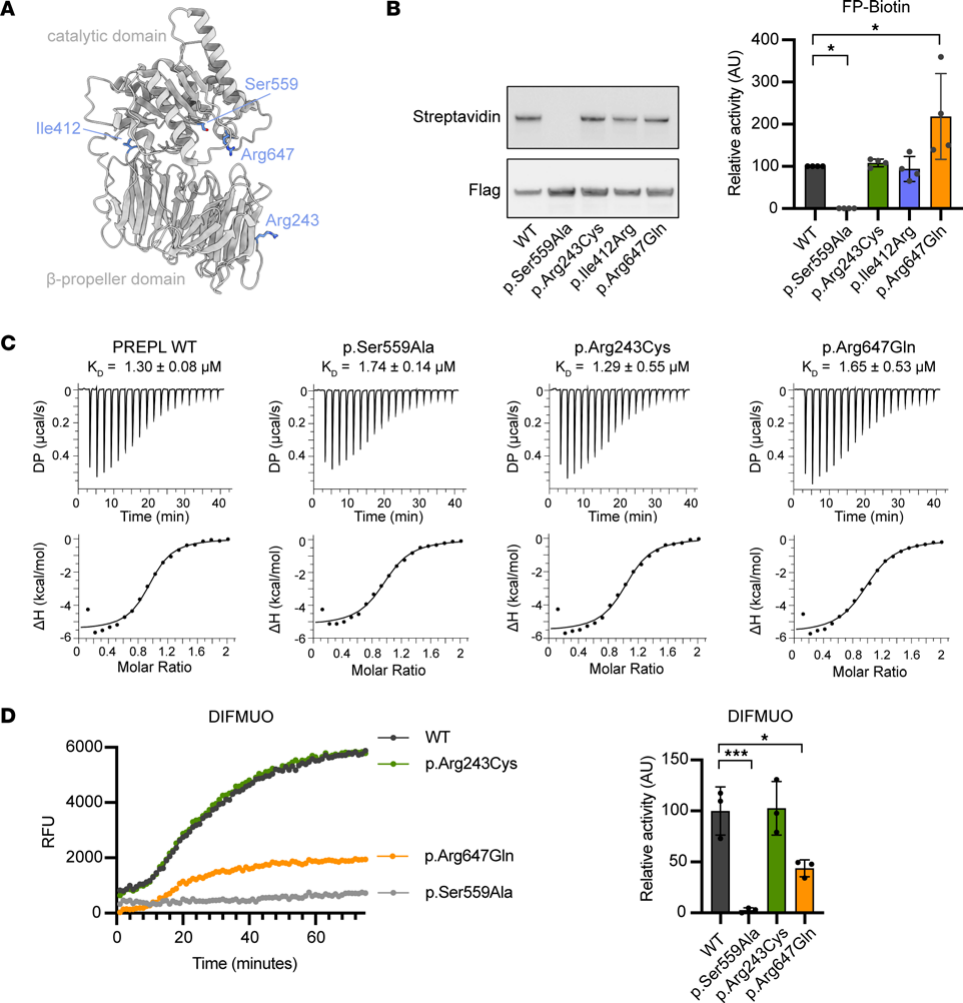
CMS22 patient PREPL variants remain catalytically active. (**A**) The 3 CMS22 mutants and the catalytically inactive PREPL p.Ser559Ala mapped on the PREPL structure. (**B**) Catalytic activity using FP-Biotin activity-based probe binding. Blot: Streptavidin = FP-biotin signal, Flag = PREPL signal. Graph: quantitative reactivity of WT and variant PREPL to the FP-biotin probe normalized to total PREPL abundance (*n* = 4) (**C**) Isothermal titration calorimetry measurements of the binding of inhibitor 8 to WT, p.Ser559Ala, p.Arg243Cys, and p.Arg647Gln PREPL. Shown are single representative traces and each stated *K*_D_ value is the mean from *n* = 3 technical replicates. (**D**) Representative graph of DIFMUO cleavage by WT and variant PREPL and calculated relative activity (*n* = 3). RFU, relative fluorescence units. Statistical analysis was performed using 1-way ANOVA. Significance levels are shown as *P ≤ 0.05 **P ≤ 0.01, ***P ≤ 0.001, and ****P ≤ 0.0001.

These patients with CMS22 have at least 1 *PREPL* allele that contains a missense variant (p.Arg243Cys, p.Ile412Arg, p.Arg647Gln), located in the catalytic domain (Ile412 and Arg647) or the propeller domain (Arg243) of the PREPL protein (Figure 1A). In order to provide a rational explanation for why these variants result in CMS22, we first investigated if they impair the catalytic activity, since all patients identified so far have a complete loss of PREPL activity. The 3 CMS22 mutants, WT PREPL, and the catalytically inactive PREPL p.Ser559Ala (Figure 1A) were produced in transiently transfected HEK293T cells and were recombinantly expressed in bacterial cells. Recombinant WT PREPL, p.Arg243Cys, p.Arg647Gln mutants and the catalytically inactive p.Ser559Ala variant were soluble, while p.Ile412Arg PREPL was aggregated in vitro and could not be purified from bacteria. Transient expression of p.Ile412Arg PREPL in HEK293T cells was normal.

Using the serine hydrolase-specific activity-based probe (FP-biotin) we found that all 3 PREPL variants (p.Arg243Cys, p.Ile412Arg, p.Arg647Gln) were able to react with the probe, suggesting that the variants remain catalytically active (Figure 1B). Further, we observed that PREPL p.Arg647Gln (218.5 ± 101.6) had an increased affinity to the FP-biotin probe compared with WT (100 ± 0.0). The replacement of the positively charged Arg by the polar Gln is likely to facilitate the hydrophobic linker of FP-biotin, making this PREPL variant more reactive with the FP-biotin probe compared with WT. In addition, we found that the c.1978_1981/p.Leu660Argfs*22 deletion identified in patient 2 completely abolished the FP-biotin binding capacity of PREPL (Supplemental Figure 1; supplemental material available online with this article; https://doi.org/10.1172/jci.insight.179276DS1).

To substantiate these results, we evaluated the binding dynamics of inhibitors to PREPL using Isothermal Titration Calorimetry (ITC). We tested the binding of inhibitor 8 (1-isobutyl-3-oxo-3,5,6,7-tetrahydro-2H-cyclopenta[c]pyridine-4-carbonitrile), a known inhibitor of PREPL (30), to the CMS22 PREPL variants. We found that WT PREPL, PREPL p.Arg243Cys, PREPL p.Arg647Gln, and the catalytically inactive PREPL p.Ser559Ala bind inhibitor 8 with similar affinity, with an approximate value of 1 μM (Figure 1C). This suggests that the binding between the protein and this inhibitor is dependent on the binding pocket and is independent of the catalytic Ser559 residue. Moreover, binding remains unaffected by the variants, which further supports the idea that the catalytic binding pocket is not affected by the variants.

FP-biotin and inhibitor 8 binding only mimic the first steps of the PREPL enzymatic mechanism, i.e., formation of the Michaelis complex and — for FP-biotin — formation of a stable tetrahedral intermediate, but not the second aqueous hydrolysis step. Therefore, we also tested the ability of PREPL variants to cleave the fluorescent ester substrate 6,8-Difluoro-4-Methylumbelliferyl octanoate (DIFMUO) (31). This substrate could be cleaved by WT PREPL but not by PREPL p.Ser559Ala (Supplemental Figure 2). The substrate cleavage efficiency by WT PREPL was 50% compared with Acyl protein thioesterase 1(APT1) (Figure 1D and Supplemental Figure 2). Moreover, we evaluated the cleavage of DIFMUO by CMS22 PREPL variants, where we saw that PREPL p.Arg243Cys (102.6 ± 26.2) had fully conserved catalytic activity, while PREPL p.Arg647Gln (43.8 ± 8.3) showed a 2-fold decrease in substrate cleavage activity compared with the WT (100.0 ± 23.6) (Figure 1D). The proximity of the p.Arg647Gln variant to the catalytic site could negatively impact the cleavage of this synthetic substrate.

The 3 point variants described above are the first missense variants described in patients with CMS22, complementing the deletions, nonsense, and frame-shift variants. Since all 3 variants have retained, at least in part, their hydrolytic activity, we selected 18 *PREPL* missense variants from the ClinVar database. The selection was based on their location in PREPL, thereby covering the whole protein, as well as positive selection if the variant was detected in a children’s hospital (32). From these, 8 reside in the β-propeller domain and 10 in the catalytic domain (Supplemental Figure 1, A–C). Using the FP-biotin activity-based probe assay, we found that none of the variants in the β-propeller domain had an effect on ABP binding. Of the 10 variants located in the catalytic domain, 3 significantly decreased FP-biotin binding (p.Tyr479Cys, p.Arg510Gln, p.Ala560Pro) while 1 significantly increased FP-biotin binding (p.Glu605Lys). Ala560, but also Tyr479 are in close proximity with the catalytic Ser559, which explains the negative impact of the variants on the ABP binding. Arg510 is localized at a distance from the catalytic triad residues, potentially explaining its less effective decrease in ABP binding compared with the p.Tyr479Cys and p.Ala560Pro mutants. In conclusion, 15 out of the 18 tested variants in ClinVar have normal ABP binding. In the absence of case reports from the corresponding patients, we cannot determine whether or not these are potential patients with CMS22, but it highlights the possibility of false negatives if only ABP binding is used for differential diagnosis.

Moreover, since several premature stop codons have been found in patients with CMS22, we created 4 early stop codons at aa687, aa709, aa719, and aa724 to determine the minimal sequence for catalytic activity (Supplemental Figure 1, A–C). Stop codons at aa719 and aa724 did not affect catalytic activity, while stop codons at aa687 and aa709 deactivated PREPL. It is worth noting that aa687 and aa709 reside in the loop preceding the last α helix and on the helix itself, respectively, within the PREPL structure. A stop codon variant at these sites could disrupt the secondary structure of PREPL, rendering it catalytically inactive.

### CMS22 PREPL variants display similar stability to the WT protein.

Since the catalytic activity of CMS22 variants is conserved or slightly affected (in the case of p.Arg647Gln), we next sought to investigate the protein stability of these PREPL variants in vitro, to test if the CMS22 phenotype may be a result of protein instability induced by the variants.

**Figure 2:**
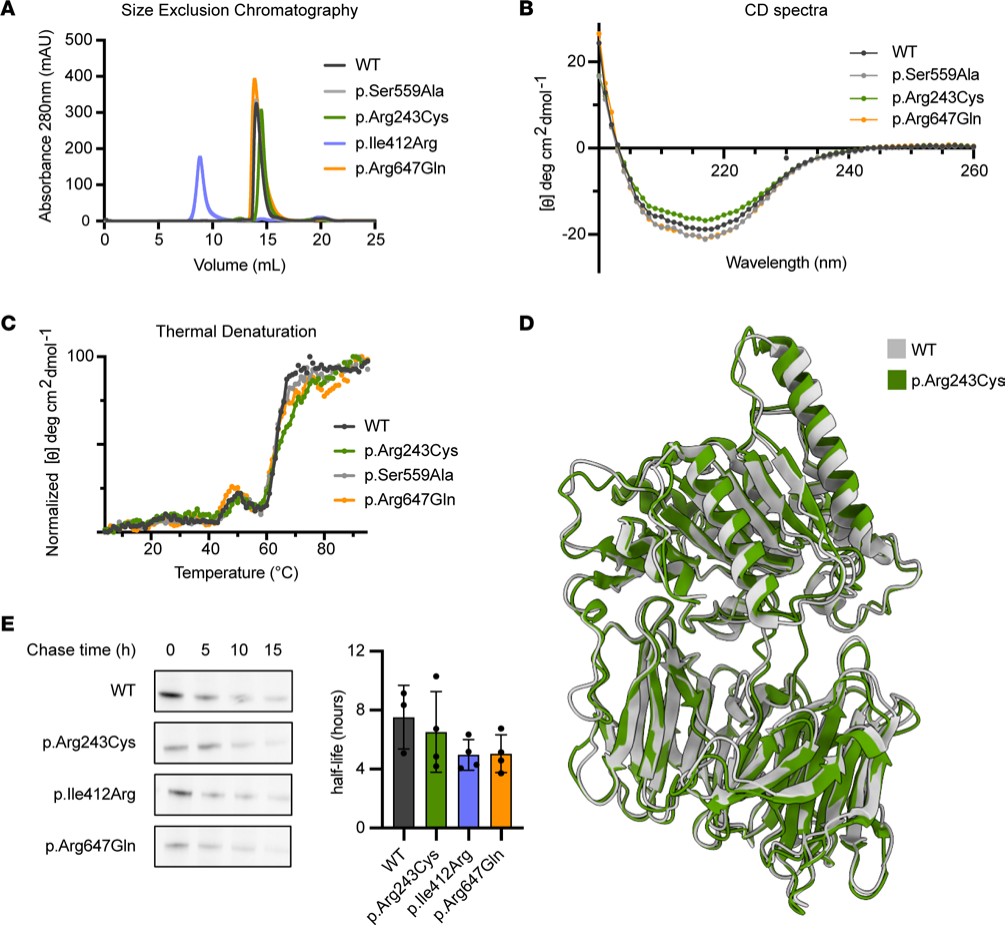
Protein stability of CMS22 patient PREPL variants. (**A**) Recombinant p.Arg243Cys, p.Arg647Gln mutants and the catalytically inactive p.Ser559Ala PREPL variant are soluble and monomeric by size exclusion chromatography. Recombinant p.Ile412Arg PREPL mutant aggregates in vitro, eluting in the void volume (8 mL). (**B**) CD spectra of CMS22 mutants, which share the same secondary structure as the WT protein. (**C**) Normalized thermal denaturation curves monitored by measuring the CD signal at 217 nm across a temperature range from 4°C to 95°C. The melting temperature (T_m_) of PREPL WT and the p.Ser559Ala and p.Arg647Gln mutants is calculated to be 63.5°C, while the melting temperature of p.Arg243Cys is 61.5°C. The unfolding process of all PREPL variants displays a 2-step pattern, with the first unfolding step occurring at approximately 50°C. (**D**) Structure of PREPL p.Arg243Cys mutant solved by cryo-EM in the open conformation, sharing the same fold as the WT protein. (**E**) Protein halflife measured by pulse chase. Representative blot of PREPL abundance after 0, 5, 10, and 15 hours of chase and quantified halflife time for WT and variant PREPL. (*n* = 3) Statistical analysis was performed using 1-way ANOVA.

Our results demonstrate that recombinant p.Arg243Cys and p.Arg647Gln PREPL mutants and the catalytically inactive p.Ser559Ala variant were soluble and displayed similar elution profiles to the WT protein by size exclusion chromatography, showing they are also monomeric (Figure 2A). As already mentioned, p.Ile412Arg PREPL was aggregated in vitro and could not be purified. We next assessed the folding and thermal stability of PREPL mutants by circular dichroism (CD) spectroscopy. p.Arg243Cys, p.Arg647Gln, and p.Ser559Ala PREPL share the same secondary structure as the WT protein (Figure 2B). They all exhibited similar thermal stability (Figure 2C), with the melting temperature (T_m_) of PREPL WT and the p.Ser559Ala and p.Arg647Gln mutants calculated to be 63.5°C, while the melting temperature of p.Arg243Cys was 61.5°C. Their unfolding process displayed a 2-step pattern indicative of the presence of 2 domains as expected: the helical catalytic domain and the β-propeller comprised of β sheets.

To investigate the potential impact of the patient variants on PREPL conformation we solved the structure of p.Arg243Cys mutant using cryoelectron microscopy (Figure 2D). This PREPL mutant was chosen due to its ability to be purified to higher concentrations compared with the other PREPL variants and its stable behavior on the cryo-EM grids. The resulting map was resolved at a resolution of 4.01 Å (using the FSC 0.143 criterion) (Supplemental Figure 3). The structure of this CMS22 PREPL mutant shows that it is in an open conformation, highly similar to the WT protein solved by crystallography (PDB ID: 7OBM) (Figure 2D), with a root mean square deviation (Ca) value of 0.980 Å. Therefore, this structural study shows that the Arg243Cys variant did not affect the ternary structure of PREPL.

Next, we sought to evaluate protein halflife of the PREPL mutants in vitro in order to evaluate if the proteins are not subject to early degradation. Therefore, we performed 35S methionine labelling of HEK293T cells and transiently transfected with the PREPL variants to determine their halflife. We established that WT PREPL has a halflife of 7.5 (± 2.2) hours (Figure 2E). The halflife of the patient PREPL variants were all slightly shorter, albeit nonsignificantly: PREPL p.Arg243Cys 6.5 (± 2.7) hours, PREPL p.Ile412Arg 5.0 (± 1.0) hours and PREPL p.Arg647Gln 5.1 (± 1.3) hours. Overall, these results show that the variants have limited or no effect on the stability of PREPL.

### Missense variants in PREPL disrupt protein-protein interactions.

**Figure 3:**
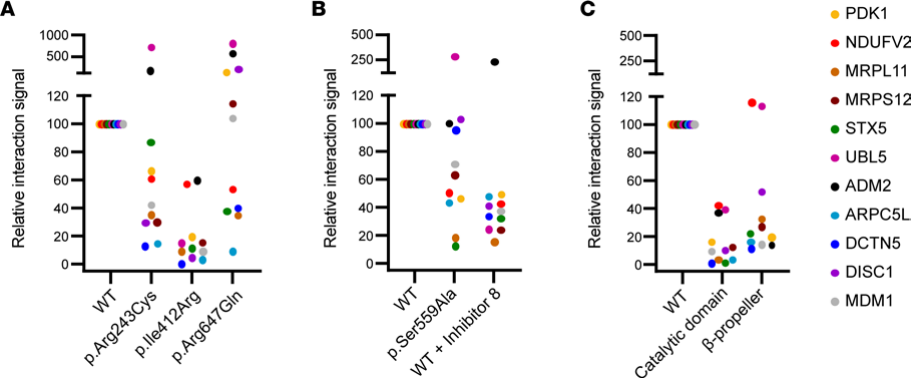
Overview of protein-protein interactions from 11 target proteins with PREPL variants. Protein-protein interaction scores with 11 previously identified PREPL interactors were determined using MAPPIT and relative interaction signals plotted for (**A**) WT PREPL, and PREPL variants p.Arg243Cys, p.Ile412Arg, and p.Arg647Gln (**B**) WT PREPL and inactivated PREPL p.Ser559Ala an WT PREPL inactivated by addition of 50 μM total concentration of inhibitor 8 (**C**) WT PREPL and the catalytic domain or β-propeller domain separately.

Recent findings suggest that protein-protein interactions are pivotal for the function of PREPL and that some of these interactions are independent of catalytic activity (15, 20, 21). Therefore, we evaluated if the CMS22 patient variants have an effect on the interactome of PREPL. Using the mammalian protein-protein interaction trap (MAPPIT), protein-protein interaction scores were measured between WT and CMS22 PREPL mutants and 11 previously identified PREPL interactors (Figure 3 and Supplemental Figure 4). These 11 interactors were selected from the previously identified list of 250 interactors on the basis of a high interaction score and divided between cell compartments and cell-biological functions (15). We found that patient-derived PREPL variants had significant changes in the interaction scores with the 11 interactors relative to WT PREPL. PREPL p.Arg243Cys had 7 decreased (NDUFV2, MRPL11, MRPS12, ARPC5L, DCTN5, DISC1, MDM1) and 1 increased interaction (UBL5); PREPL p.Ile412Arg had 8 decreased interactions (NDUFV2, MRPL11, MRPS12, STX5, ARPC5L, DCTN5, DISC1, MDM1); and PREPL p.Arg647Gln had 5 decreased (NDUFV2, MRPL11, STX5, ARPC5L, DCTN5) and 3 increased interactions (UBL5, ADM2, DISC1) (Figure 3A and Supplemental Figure 4). These data indicate that the missense variants alter the interactome of PREPL. Previously, Männistö et al. (2017) suggested that catalytic activity might have a modulatory role for allowing protein-protein interactions of PREP (22). Therefore, we evaluated if the catalytic activity contributes to protein-protein interactions by measuring interaction scores using a catalytically inactive PREPL p.Ser559Ala mutant and WT PREPL treated with the previously reported PREPL inhibitor 8 (Figure 3B). Indeed in both cases, overall interactions were decreased with 6 interactors (NDUFV2, MRPL11, MRPS12, STX5, ARPC5L, MDM1) for the p.Ser559Ala mutant and 8 interactors (NDUFV2, MRPL11, MRPS12, STX5, ARPC5L, DCTN5, DISC1, MDM1) for the inhibitor 8–treated WT PREPL. This indicates that the catalytic activity is a contributing factor to protein-protein interactions of PREPL. To evaluate if protein-protein interactions of PREPL are domain specific we analyzed the interactions formed by the catalytic domain and β-propeller domain independently (Figure 3C and Supplemental Figure 4). We found that the individual domains were unable to maintain protein-protein interactions similar to WT. Only 2 interactors scored positively with the β-propeller alone (NDUFV2, UBL5). This indicates that both domains in the PREPL structure are needed for full protein-protein interactions, suggesting that interaction sites span across both domains or are a result of a dynamic interplay between the 2 domains.

### PREPL displays both catalytic and noncatalytic functions in HEK293T cells.

**Figure 4:**
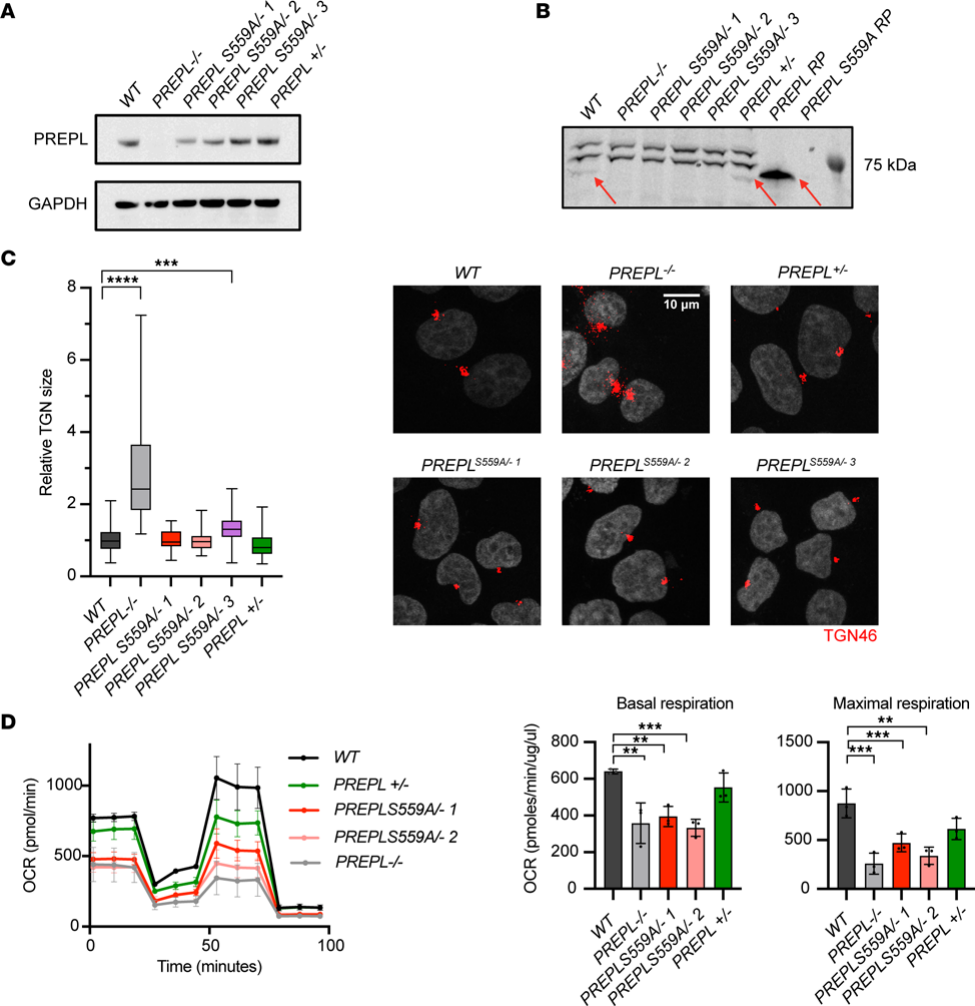
*PREPL*^S559A^ mutant cells have a normal TGN morphology and display mitochondrial respiratory dysfunction. (**A**) PREPL expression levels in 20 μg of HEK293T cell lysate from WT and PREPL variant cell lines. (**B**) PREPL activity evaluated by FP-TAMRA in lysates from WT and PREPL variant cell lines. (**C**) Evaluation of TGN size by confocal microscopy between WT and PREPL variant cell lines (*n* = 37–75). (**D**) Evaluation of mitochondria function by Seahorse cell mito stress test between WT and PREPL variant cell lines (*n* = 3). Data were analyzed by Mann-Whitney *U* test (multiple comparison corrected by Dunnett’s test). Significance levels are shown as **P ≤ 0.01, ***P ≤ 0.001, and ****P ≤ 0.0001.

Biochemical evaluation of the patient PREPL variants shows that catalytic activity is conserved but protein-protein interactions are altered. Moreover, MAPPIT evaluation shows that both domains need to be present for protein-protein interactions and that catalytic activity might also play a contributing role in the strength or nature of PREPL interactions. In order to evaluate the contribution of catalytic activity to the function of PREPL we used CRISPR/Cas9 to create 3 independent catalytically inactive PREPL p.Ser559Ala HEK293T cell lines, and 1 *PREPL*-KO HEK293T cell line. After CRISPR/Cas9, Sanger sequencing revealed that all 3 PREPL p.Ser559Ala HEK293T cell lines have 1 mutant p.Ser559Ala PREPL allele combined with 1 premature stop-codon allele. Using Western blotting, we show that PREPL was unable to bind to the fluorescent activity-based probe FP-TAMRA in all 3 PREPL p.Ser559Ala/– cell lines, thereby validating that PREPL p.Ser559Ala is catalytically inactive (Figure 4, A and B). These cell lines mimic the 2 patients with 1 null allele and 1 missense mutation (Patients 1 & 2). PREPL activity was also recorded in WT, a PREPL+/– 1 allelic expression control cell line, and recombinant expressed WT PREPL (Figure 4B, red arrows).

To evaluate the contribution of catalytic activity to the possible function of PREPL, we subjected these PREPL p.Ser559Ala/– cells lines to 2 functional tests. First, Radhakrishnan et al. (2013) have demonstrated that PREPL interacts with the μ1A subunit of AP-1 and regulates AP-1 membrane binding. PREPL-deficient fibroblasts from patients with hypotonia-cystinuria syndrome (HCS; MIM 606407) show an increased AP-1 membrane binding and present an increased TGN size. Increased AP-1 membrane binding could be rescued by overexpression of WT or p.Ser559Ala mutant PREPL, suggesting a nonenzymatic activity. We show that relative TGN size is significantly increased in PREPL –/– HEK293T cells (2.89 ± 1.45) compared with WT cells (1.00 ± 0.31) (Figure 4C). Interestingly, 2 PREPL p.Ser559Ala/– HEK293T cell lines displayed a relative TGN size similar to WT: PREPL S559A/– 1 (1.00 ± 0.25) and PREPL S559A/– 2 (1.02 ± 0.33), while PREPL S559A/– 3 (1.34 ± 0.42) had a slightly increased relative TGN size. A normal relative TGN size was also recorded in the PREPL +/– 1 allelic expression control cell line (0.87 ± 0.34). Further, addition of PREPL specific inhibitor 8 did not affect TGN morphology (Supplemental Figure 5). These data suggest that TGN morphology is regulated by PREPL in a catalytically independent manner and that monoallelic PREPL expression is sufficient to maintain normal TGN morphology.

Moreover, PREPL is involved in mitochondrial protein translation and mitochondrial respiration (15, 33). Therefore, we evaluated mitochondrial respiration in our HEK293T cell lines using a Seahorse XFp extracellular flux analyzer (Figure 4D). We found that basal mitochondrial respiration was significantly reduced in PREPL–/– HEK293T cells compared with WT while respiration remained normal in the PREPL +/– line. Moreover, reduced basal respiration was recorded in PREPL p.Ser559Ala/– 1 and PREPL p.Ser559Ala /– 2 HEK293T cells lines compared with WT cells. PREPL p.Ser559Ala /– 3 was not tested. Similarly, maximal respiration was significantly reduced in PREPL–/– and PREPL p.Ser559Ala /– 2 HEK293T cells compared with WT. These results suggest that catalytic activity is pivotal in the function of PREPL in the mitochondria.

## Discussion

In this study, we describe a new category of CMS22 variants, which should be considered in the differential diagnosis of congenital myasthenia. We studied 3 CMS22 patients that harbor compound heterozygous or homozygous missense variants in *PREPL* that do not block the enzymatic activity of PREPL in vitro. These patients present the full CMS22 phenotype and share the hallmark CMS22 phenotypes of neonatal hypotonia and growth delay. As CMS22 is a recessive disorder, these missense variants are predicted to be detrimental to the function of PREPL, thereby eliciting the CMS22 phenotype. However, all 3 PREPL variants (p.Arg243Cys, p.Ile412Arg, and p.Arg647Gln) react with the FP-biotin ABP. This shows that CMS22 diagnosis should not depend only on a negative FP-biotin test, thereby challenging the conventional diagnostic criteria that relies on a negative PREPL ABP binding test from a blood sample (7). Moreover, PREPL p.Arg243Cys from patient 1 retained normal catalytic activity using a fluorescent substrate active in vitro while the cleavage capacity of PREPL p.Arg647Gln was reduced 2-fold.

**Figure 5:**
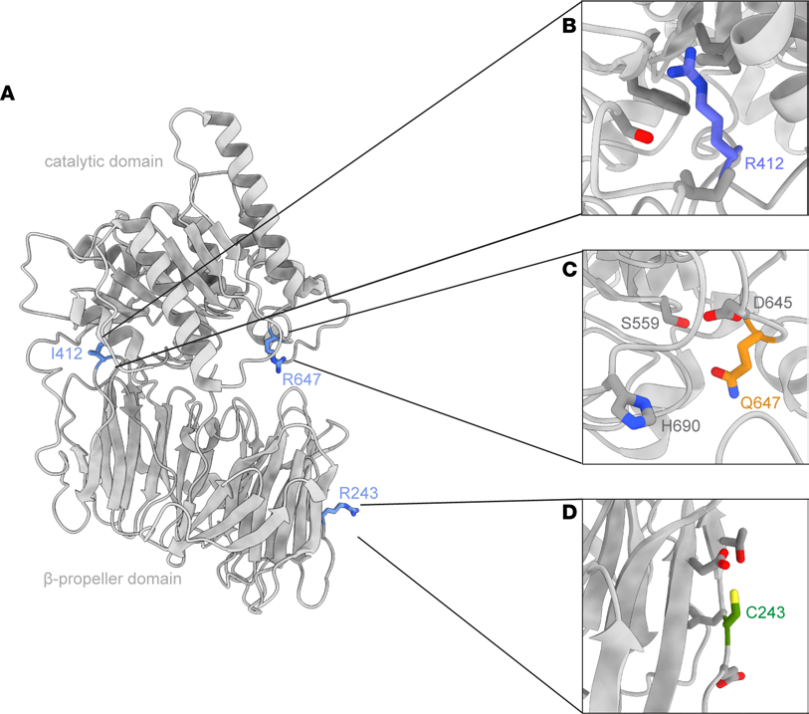
Location of CMS22 PREPL variants on the PREPL structure. (**A**) Location of the CMS22 patient mutations within the PREPL crystal structure (PDB: 7OBM). (**B**) Arg412 is located on the β-propeller in the vicinity of the hydrophobic residues Val498, Leu504, Ser441, Ile437, and Phe461. (**C**) Gln647 is on the catalytic domain close to the catalytic triad residues Ser559, Asp645, and His690. (**D**) Cys243 and nearby PREPL residues are found on an exposed surface of the β-propeller domain.

Two patients examined in this study were compound heterozygous with a frameshift variant leading to a premature stop codon on one allele and a missense variant on the other. In both cases, the carboxyterminal deletions (p.Leu597 and p.Leu660) are predicted to lead to a catalytically inactive allele since we demonstrated that truncation of PREPL at Glu687 already inactivates PREPL. These stop codon mutations disrupt the secondary structure of PREPL by cutting the last helix of the catalytic domain short and lead to a catalytically inactive PREPL. Examining further the PREPL structural data from crystallography and cryo-EM can help us rationalize the effects of mutations found in patients. Arg243 is positioned on PREPL in the loop connecting the β-11 and β-12 sheets of the third blade of the propeller and is not conserved across the homologous PREP, OpdB, and AAP. This residue resides in an exposed region on the protein surface, where neither the original arginine nor the cysteine undergoes any interactions with neighboring residues (Figure 5D and Supplemental Figure 6A). Based on its location in the PREPL structure, it is not surprising that the variant retains catalytic activity but, instead, may affect protein-protein interactions mediated by the β-propeller. Next, Ile412 is on the β-propeller loop that links the β-26 and β-27 sheets of the last blade, just before the hinge loop (residues 424–440) that connects the 2 PREPL domains (Figure 5A) and does not display conservation within homologous protein sequences. Ile412 faces the catalytic domain of PREPL and resides in a hydrophobic region of the protein formed by Val498, Leu504, Ser441, Ile437, and Phe461 (Supplemental Figure 6B). The substitution of the hydrophobic isoleucine with the positively charged arginine could potentially disrupt this hydrophobic pocket within PREPL (Figure 5B). This could consequently impact the interaction between the 2 domains, potentially leading to protein instability and causing aggregation in vitro. Lastly, Arg647 is a highly conserved residue across PREPL homologues and is positioned on a loop within the catalytic domain of PREPL, in close proximity to the catalytic triad residues (Figure 5C). It has been previously proposed to be part of a putative lipid-binding pocket in PREPL (15). In PREPL homologous structures, Arg647 appears in 2 different conformations, making salt bridges with nearby residues in order to stabilize either the active or inactive form (Supplemental Figure 6, C–E). Hence, mutation of Arg243 to Gln could disrupt these interactions, affecting the protein’s catalytic activity. How big the impact is on physiological substrates remains to be established.

We showed that all CMS22 PREPL variants have similar thermostability and halflife as the WT. Moreover, as suggested by the p.Arg243Cys cryoEM structure that we were able to solve, they share the same ternary structure. Nevertheless, the interactome of the PREPL variants was severely affected. We found that the patient missense variants p.Arg243Cys and p.Ile412Arg significantly reduced the interactions with (almost) all interactors tested. These interactors were selected from a previously identified list (15) based on high interaction scores in the MAPPIT screen and by choosing a pool of interactors that localize in different cellular locations (mitochondria and cytosol). Ipso facto, loss of interactions is the likely cause for loss of function of this allele in patients 1 (p.Arg243Cys) and 2 (p.Ile412Arg). Remarkably, p.Arg647Gln showed reduced interactions with about half the interactors but increased interactions with the other half. The increased reactivity with FP-biotin but decreased cleavage of the DIFMUO ester substrate are consistent with a model in which this variant results in reduced activity and altered specificity ultimately leading to a null allele. Further in vitro studies have highlighted the importance of both the catalytic and β-propeller domains to be together in order to facilitate protein-protein interactions and the necessity of functional catalytic activity, hinting at a collaborative function between catalytic activity and protein-protein interactions.

To shed more light on the collaborative function between catalytic activity and protein-protein interactions, we evaluated the effect of catalytic inactivation (p.Ser559Ala) on the cell-biological function of PREPL. Mitochondrial respiratory activity is dependent on catalytic activity of PREPL, suggesting that substrate cleavage is crucial for mitochondrial regulation. Since the missense variants in patients 1 (p.Arg243Cys) and 2 (p.Ile412Arg) are not altering the catalytic activity of PREPL, we expect mitochondrial dysfunction to be due to alterations of protein interactions. Previously, Radhakrishnan et al. have shown that TGN morphology is regulated by PREPL independently of its catalytic activity, through protein-protein interactions with the μ-1A subunit of AP1. We created a more robust and high throughput method to measure TGN size with increased sample size and used endogenously inactivated PREPL p.Ser559Ala in order to validate that AP1 recycling does not require the catalytic activity of PREPL. Together, these functional assays highlight the importance of catalytically independent protein-protein interactions for the physiological function of PREPL.

**Figure 6:**
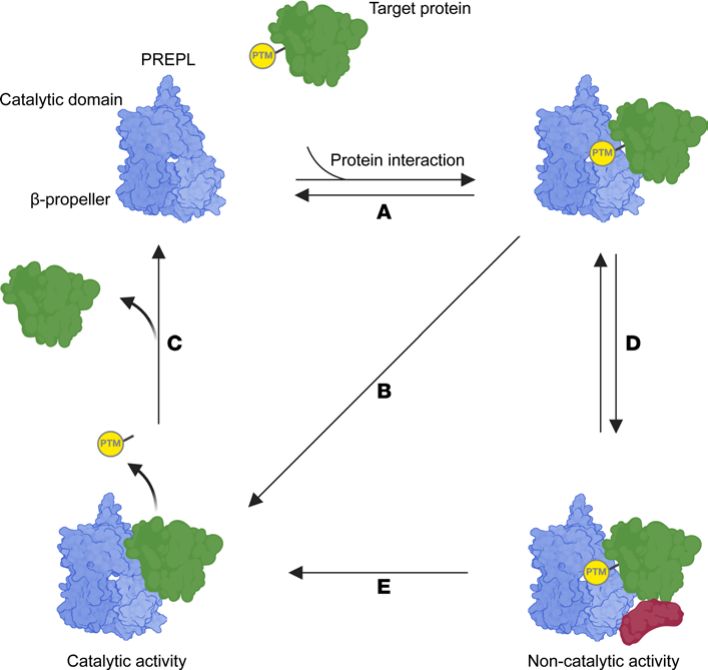
A proposed functional cycle for PREPL incorporating catalytic and noncatalytic activity. Based on our in vitro results, we propose a functional cycle for PREPL where PREPL first binds a target protein (**A**), potentially facilitated by a PTM on the target protein. Next, it can follow 2 paths: (a) PREPL cleaves a PTM from the target protein (**B**), breaking the complex (**C**) and thereby completing its physiological function. Alternatively, (b) the protein interaction can continue independent of catalytic activity and could expand to bigger complexes (red protein) (**D**) (noncatalytic function). These complexes can dissociate without the need for catalytic activity or by the removal of a potential PTM by PREPL (**E**).

Based on our results, we propose a new model for PREPL function (Figure 6). In this model, PREPL first binds a target protein (Figure 6A). This interaction might be facilitated or enhanced by alignment of a PTM on the target protein within the substrate binding pocket of PREPL. Afterward, 2 different paths are possible: (a) PREPL cleaves a PTM from the target protein (Figure 6B), breaking the interaction (Figure 6C) and thereby completing its catalytic function. Alternatively, (b) the interaction between PREPL and the target protein can continue independently of the catalytic activity and facilitate the formation of bigger complexes (noncatalytic function) (Figure 6D). These complexes can dissociate without the need for catalytic activity or by the removal of a potential PTM by PREPL (Figure 6E). In this functional cycle, and in contrast with the previously suggested model for PREP (22), catalytic activity alone does not modulate protein-protein interactions but both catalytic and noncatalytic activities of PREPL coexist to fulfill the physiological functions of PREPL. We hypothesize that the PTM in this model could be palmitoylation, as PREPL is inhibited by palmostatin M, and palmitoylation is increased in PREPL KD N2A cells (15, 19).

In our MAPPIT assay, 4 interactors are located in mitochondria (PDK1, NDUFV2, MRPL11, and MRPS12), and PREPL KO is known to cause mitochondrial dysfunction. Moreover, PTMs like palmitoylation are key regulators of mitochondrial function. Mutations causing loss of interaction between PREPL and these mitochondrial proteins could lead to the accumulation of PTMs on these interactors, which may result in mitochondrial dysfunction. Similarly, loss of PREPL is known to cause defects in the secretory pathway. We hypothesize that aberrant PTMs on interactors DCTN5, ARPC5L, DISC1, and STX5 could be the molecular cause of these defects. Consequently, the next steps in this research would be to evaluate the effects of PREPL in the PTMs of its interactors and whether these are mediated by catalytic or noncatalytic mechanisms.

Although the in vitro data support the model described in Figure 6, we cannot fully exclude that reduced expression or instability of PREPL in vivo causes or contributes to the pathophysiology observed in the patients.

In conclusion, in this study we have presented what we believe to be a new category of CMS22 loss of function variants. This category of variants still has PREPL hydrolytic activity in vitro but impaired protein-protein interactions, leading to impaired function and the CMS22 phenotype. Our previously developed functional blood assay (7) remains useful for identifying deleterious variants in intronic or regulatory genomic regions, but exonic variants require further scrutiny. In addition to substrate or ABP reactivity, protein-protein interactions should be considered.

## Methods

### Sex as a biological variant.

This study includes 2 male patients and 1 female patient. The genetic variants were studied in an equal manner and we did not consider sex as a biological variant regarding the effect of the genetic variants on the function of PREPL.

### Bacterial protein expression and purification.

The human PREPL_S_ (Uniprot sequence Q4J6C6, residues 90–727) expression plasmid carries the gene for the PREPL_S_ protein isoform, with an N-terminal 8×Histidine tag and an MBP (Maltose-binding protein) tag, followed by a TEV (Tobacco Etch Virus) cleavage site. PREPL_S_ was cloned into the bacterial expression vector pVP68K and mutations were introduced using the QuikChange II site directed mutagenesis kit (Stratagene) and confirmed by direct Sanger sequencing. The plasmids were transformed into Rosetta(DE3)pLysS cells (Sigma-Aldrich). Protein expression was induced with isopropyl b-D-1- thiogalactopyranoside (IPTG; 0.4 mM) when cells reached an optical density (OD at 600 nm) of 0.9, with a subsequent growth over night at 20°C. Cell pellets were resuspended in lysis buffer (500 mM NaCl, 50 mM Hepes pH 7.4 [Chemie Brunschwig AG], 1 mM TCEP, 5% glycerol [Merck]) and were lysed by sonication. After centrifugation at 20,000*g* for 60 minutes at 4˚C, the supernatant was filtered and applied to 2 HisTrap HP columns (GE Healthcare). After elution of recombinant PREPL with a continuous gradient over 40 column volumes of elution buffer (500 mM NaCl, 50 mM Hepes pH 7.4 [Chemie Brunschwig AG], 1 mM TCEP, 5% glycerol [Merck], 500 mM imidazole [Merck]), pure fractions were buffer exchanged into lysis buffer to remove imidazole, using a HiPrep Desalting column (GE Healthcare). The N-terminal purification tags were cleaved by overnight incubation with TEV protease. Subsequently, the cleaved tags and TEV protease were removed with another His affinity chromatography and the flowthrough was collected, concentrated, and additionally purified by Size Exclusion Chromatography (HiLoad 16/600 Superdex 200 pg, Cytiva) in lysis buffer. Pooled fractions were concentrated to approximately 10 mg/mL using a MWCO concentrator (Sigma-Aldrich) with a cutoff of 30 kDa and stored at –20°C.

### Cell culture, transient transfection, and immunoprecipitation.

Generation of FLAG-tagged PREPL constructs for overexpression in mammalian cells has been described before (12). HEK293T cells were grown in DMEM supplemented with 10% FCS at 37°C, 5% CO2 and transfected using the Xtreme Gene 9 transfection reagent (Merck) according to the manufacturer’s protocol. 24 hours after transfection, cells were lysed by passaging through a 26-gauge needle. Cell debris was removed by centrifugation at 16,000 *g* for 10 minutes. FLAG-tagged proteins were immunopurified using preformed complexes of anti-FLAG M2 antibody (Sigma-Aldrich) and protein G Sepharose (GE healthcare).

### Metabolic labelling.

Metabolic labeling was performed as previously described (34). Briefly, WT and mutated (p.Arg243Cys, p.Ile412Arg and p.Arg647Gln) PREPL pcDNA3 plasmids were transiently transfected into HEK293T cells after which cells were starved for 1 hour in methionine-free RPMI (Thermo Fisher Scientific) and subsequently pulsed for 30 minutes in starvation medium supplemented with 200 μCi/mL EasyTagTM EXPRESS 35S protein labeling mix (Perkin Elmer). Cells were chased in RPMI supplemented with 0.4 mM methionine for 0, 6, 12,and 24 hours and subsequently lysed in 50 mM Tris pH 7.4, 150 mM NaCl, 1% Triton, 1 mM EDTA and 1 × cOmpleteTM mini protease inhibitor cocktail (Roche). PREPL proteins were immunoprecipitated using anti-FLAG M2 antibody (Sigma-Aldrich) and separated by SDS-PAGE. Radioactive signal was amplified with NAMP100V (Sigma-Aldrich) and the gel was dried using a gel dryer (Biorad). Signal was captured using a TyphoonTM FLA 9500 biomolecular imager.

### Circular dichroism spectroscopy.

Circular Dichroism Spectra of the PREPL variants (at 10 μM in 100 mM NaCl, 20 mM NaH_2_PO_4_ pH 7.4 and 1 mM TCEP) were collected with a Chirascan V100 (AppliedPhotophysics), using a 1 mm path length cuvette, at 20°C, in the 200–260 nm range. Thermal denaturation experiments were performed by heating the sample from 4°C to 95°C and collecting CD data at 217 nm every 2°C, with a temperature ramp speed of 1°C/min and an equilibration time of 15 seconds.

### Isothermal titration calorimetry.

Isothermal titration calorimetry (ITC) experiments were performed on a MicroCal PEAQ-ITC (Malvern Panalytical). PREPL proteins used for ITC measurements were in a buffer containing 300 mM NaCl, 20 mM Hepes pH 7.4 and 1 mM TCEP. Inhibitor 8 (1-isobutyl-3-oxo-3,5,6,7-tetrahydro-2H-cyclopenta[c]pyridine-4 carbonitrile) (Thermo Fisher Scientific) was synthesized and delivered as lyophilized powder (Vitas M Chemical) and was dissolved in 100% DMSO at a 100 mM concentration. Before measurement, PREPL proteins were extensively dialyzed against the aforementioned buffer, and the ligand was diluted in the same dialysis buffer to prevent buffer mismatch, to a final concentration of 1 mM at 1% DMSO. Experiments consisted of titrations of a single 0.4 μL injection followed by 19 injections of 2 μL of titrant (inhibitor 8) into the cell containing PREPL protein at a 50 μM concentration. Experiments were performed at 25°C in triplicate (*n* = 3), with injection duration of 4 seconds, injection spacing of 150 seconds, stir speed of 750 RPM, and reference power of 7 μcal/s. Data were processed and plotted using MicroCal PEAQ-ITC Analysis Software and fitted to a 1-site binding model.

### Cryo-EM data acquisition.

PREPL Arg243Cys was buffer exchanged into EM buffer (150 mM NaCl, 20 mM Hepes pH 7.4, 1 mM TCEP) by gel filtration chromatography using the Superdex 200 Increase 10/300 GL (Cytiva) and concentrated to 13 mg/mL using a 30 kDa cutoff MWCO concentrator (Millipore). Cryo-EM grids were prepared with an EM GP2 Automatic Plunge Freezer (Leica Microsystems). UltrAuFoil R1.2/1.3 Holey Gold foil on Gold 300 mesh grids from Quantifoil were glow-discharged for 90 seconds at 15 mA using the GloQube Plus Glow Discharge System from Quorum. 4.0 μL of PREPL Arg243Cys at 13 mg/mL was applied to the glow-discharged grids, back-blotted for 4 seconds under blot force 10 at 95% humidity and 4°C in the sample chamber and plunge-frozen in liquid ethane cooled by liquid nitrogen. Grids were loaded in a TFS Glacios microscope (200 kV) and screened for particle presence and ice quality, and the best grids were transferred to TFS Titan Krios G4. Cryo-EM data was collected using TFS Titan Krios G4 transmission electron microscope (TEM), equipped with a Cold-FEG on a Falcon IV detector in electron-counting mode (EER). Falcon IV gain references were collected just before data collection. Data was collected using TFS EPU software package with aberration-free image shift protocol (AFIS), recording 9 micrographs per hole. Movies were recorded at nominal magnification of 250,000×, corresponding to the 0.3084 Å pixel size at the specimen level, with defocus values ranging from –1.5 to –2.5 μm. Exposure time was adjusted to 1 second per movie resulting in a total dose of 60 e-/Å^2^. In total, 10,745 micrographs in EER format were collected.

### Cryo-EM data processing.

During data collection, initial processing was performed using CryoSPARC live v3.2.0 (35) for assessing data quality. The obtained ab-initio structures from on-the-fly processing were used for particle picking and template generation. Motion correction was performed on raw stacks without binning, using the CryoSPARC Patch motion correction implementation. A total of 1,059,238 particles were template-based automatically picked and particles were binned by a factor of 3 during extraction. Two rounds of 2D classification were performed to remove bad particle picks, resulting in a set of 251,714 particles. Selected particles resulting from the 2D classification were used for ab initio reconstruction and subsequent nonuniform refinement and local refinement. After refinement, 137,436 particles were reconstructed into a 3D map of 4.01 Å resolution. The reported resolution is based on the gold-standard Fourier shell correlation 0.143 criterion. Local-resolution variations were estimated using CryoSPARC.

### Model building and refinement.

The PREPL p.Arg243Cys model was generated using the crystal structure of PREPL WT (PDB 7OBM) (15), by mutating the Arg243 to Cys in UCSF ChimeraX (36). The generated mutant model was fit into the cryo-EM map with UCSF ChimeraX and was manually adjusted using Coot (37). The final model was generated after iterative cycles of manual model building in Coot and relaxed refinement in Rosetta (38). The areas corresponding to the N terminal loop of PREPL from residues 95–111 were not built, since the electron density at this area was not well defined. The same applies for the loop areas corresponding to amino acids 134–139 and 687–693. Sidechains of surface residues distributed all over were deleted when their position was not well defined by the electron density. The final model was validated using MolProbity (39) and EMRinger (40) (Supplemental Table 1). Figures were prepared using UCSF Chimera, UCSF ChimeraX, and PyMOL.

### Activity-based probes assay.

WT PREPL and PREPL variants (p.Arg243Cys, p.Ile412Arg, and p.Arg647Gln) were transiently transfected in HEK293T cells. Subsequently, cells were lysed and recombinant WT PREPL and PREPL variants were immunoprecipitated using antibodies directed against the FLAG-tag. ABP-labeling was performed by incubating immunoprecipitated proteins for 30 minutes at 37°C with 1.5 μM FP-biotin in 50 mM phosphate buffer (pH 8) supplemented with 10 mM EDTA and 2 mM DTT. FP-biotin binding to PREPL occurs by covalent binding to the serine 559 residue of the catalytic triad only if it is in an active conformation. The reaction was stopped by the addition of SDS-PAGE loading buffer and samples were separated by SDS-PAGE. Covalent FP-biotin binding, indicative for serine hydrolase activity, was detected using a Streptavidine-HRP antibody (Dako). Membranes were subsequently stripped and relabeled with an anti-FLAG M2 antibody (Sigma-Aldrich F3165) to detect the presence of PREPL protein for normalization

### Fluorescent substrate cleavage assay.

Substrate cleavage assays were performed using 6,8-Difluoro-4-Methylumbelliferyl octanoate (DIFMUO), an octanoate esterified to a DIFMU fluorophore (31) DIFMUO substrate cleavage was performed using bacterially expressed PREPL variants as described above. Cleavage assays were performed in triplicate and each reaction consisted of 110 μL of reaction mixture containing 0.4 μM of protein, 0.1 mM DIFMUO, 50 mM Tris-HCl (pH 7.4), 150 mM NaCl, and 9% DMSO. Fluorescence was measured at 1 minute intervals for 100 minutes using a FLUOstar Galaxy microplate reader (BMG Labtech) at excitation and emission wavelengths of 390 nm and 460 nm.

### MAPPIT evaluation of protein-protein interactions.

MAPPIT was performed as described before (15, 41, 42). In short, HEK293T cells were plated in a 96-well plate at 10,000 cells/well. Subsequently, cells were cotransfected with 250 ng bait (PREPL or PREPL variants p.Arg243Cys, p.Ile412Arg, p.Arg647Gln), 500 ng prey (Previously identified PREPL interactors), and 50 ng pXP2d2-rPAP1-luciferase reporter plasmid using the X-tremeGENETM 9 DNA transfection reagent according to the manufacturer’s protocol. A pSEL (+2L) PREPL_S_ containing vector was used as bait and screened with a selection of previously identified interactors in a pMG1 vector. Cells were stimulated 24 hours after transfection using NeoRecormon (Roche) at 5 ng/mL in DMEM while nonstimulated cells received DMEM. Luciferase activity was measured 24 hours after stimulation using a Victor X3 plate reader (Perkin Elmer). Interactions were normalized using 2 negative controls: irrelevant bait (pSEL (+2L)) and irrelevant prey (pMG1).

### CRISPR/Cas9 genome editing.

HEK293T PREPL–KO and PREPL p.Ser559Ala mutant cells were generated using the CRISPR/Cas9 genome engineering system according to Ran et al. (43). Briefly, guide RNAs targeting PREPL were designed and cloned into a pSpCas9(BB)-2A-Puro vector (Addgene 48139). For the p.Ser559Ala cell line, an additional repair ssODN template was designed containing the desired mutation. Subsequently, cells were transfected and treated with 2 mg/mL of puromycin for 3 days to select transfected cells. Single cells were grown in order to guarantee clonality. *PREPL* KO and PREPL p.Ser559Ala were confirmed using SDS-PAGE and Sanger sequencing. Oligonucleotides are listed in Supplemental Table 2.

### FP-TAMRA activity detection of PREPL.

HEK 293T cells (WT, PREPL KO, or PREPL p.Ser559Ala) were pelleted using a benchtop centrifuge (6,000*g*, 10 minutes, 4°C). Pelleted cells were lysed by agitation in 150 μL lysis buffer (20 mM Na_2_HPO_4_, 750 mM NaCl, 0.5% Triton X-100, pH 7.4) in an Eppendorf tube. The lysates were spun down using a benchtop centrifuge (13,000*g*, 5 minutes, room temperature) to pellet debris. Total protein concentration of each lysate was determined via BCA assay. All lysates were normalized to the same protein concentration (1 mg/mL) via dilution with storage buffer (20 mM Na_2_HPO_4_, 750 mM NaCl, pH 7.4). The same storage buffer was used to make 5 μg/mL dilutions of purified PREPL proteins (WT and catalytic mutant). 60 μL of all samples (HEK lysates and purified PREPL) was transferred to fresh Eppendorf tubes and incubated with 0.6 μL of a 100 μM stock of FP-TAMRA in DMSO (final concentration of 1 μM) for 1 hour. Next, 20 μL of 4× sample buffer was added to all samples, after which they were boiled at 95°C for 2 minutes. The samples were resolved on a 12% acrylamide gel using SDS-PAGE, after which probe-labeled proteins were visualized by measuring in-gel fluorescence on a Typhoon FLA 9500 scanner with excitation at 532 nm and detection at 568 nm with the photomultiplier set at 800 V.

### Confocal microscopy.

WT- and *PREPL*-KO HEK293T cells were grown on glass coverslips coated with Poly D-Lysine (Merck A-003-E) and fixed with 4% formaldehyde. Cells were labeled with anti-TGN46 antibody (Abcam; 16059; 1:300) and Alexa Fluor-conjugated secondary antibody (Invitrogen; 21428R; 1:3000). Cells were mounted using ProLongTM Antifade Mountant + DAPI (Thermo Fisher Scientific). Confocal images were captured using the Nikon C2 confocal microscope, with 60× oil immersion optics. Images were collected using the NIS Elements software (Nikon). Quantification was performed using ImageJ software.

### Seahorse cell mito stress test.

Mitochondrial bioenergetics were analyzed by measuring oxygen consumption rates (OCR) using the Agilent XF24 extracellular flux analyzer (Seahorse Bioscience) according to the guidelines of the manufacturer. Briefly, HEK293T were seeded in XF24 cell culture plates at a density of 45,000 cells/well and 30,000 cells/well in DMEM containing 10% FCS. After 20–24 hours incubation, adherent cells were washed twice with prewarmed XF base medium supplemented with 10 mM glucose, 2 mM glutamine, and 1 mM sodium pyruvate; pH 7.4 and incubated for 1 hour at 37°C. The sensor cartridge was hydrated overnight at 37°C with XF Calibrant and calibrated using the XF24 analyzer. Oxygen consumption rates (OCRs) were measured under baseline conditions followed by the sequential addition of oligomycin, carbonyl cyanide trifluoromethoxyphenylhydrazone (FCCP), and antimycin A to injector ports A, B, and C of the cartridge, respectively. The final concentrations of the injections were 2 mM oligomycin, 0.5 mM FCCP, and 1 mM antimycin A for HEK293T and 10 mM oligomycin, 2.7 mM FCCP, and 10 mM antimycin A for skin fibroblast cells. OCRs were normalized to the protein concentrations determined by the Pierce BCA Protein assay kit (Thermo Fisher Scientific).

### Western blotting.

The PierceTM BCA Protein Assay kit (Thermo Fisher Scientific) was used to quantify protein concentrations. After denaturation, proteins were separated using SDS-PAGE electrophoresis on a 10% Bis-Tris gel and transferred to a nitrocellulose membrane. Next, membranes were blocked with 0.1 M Tris HCl pH 7.4 and 0.15 M NaCl with 0.5% blocking reagent (Roche) supplemented with 0.2% Triton X-100. Membranes were labelled with primary antibody for 1 hour at room temperature or overnight at 4°C followed by incubation with HRP-conjugated secondary antibodies (1:3000) (Dako). Membranes were developed using the Western Lightning ECL system (Perkin Elmer). The antibodies used were PREPL (Santa Cruz;393321; 1:1000) and FLAG M2 (Sigma-Aldrich;F3165; 1:10000)**.**

### Statistics.

Statistical analyses were performed using Prism GraphPad Prism 8 for Windows, GraphPad Software, www.graphpad.com Normal distribution of data was assessed with the Shapiro-Wilk test. For normally distributed data, statistical analysis was performed using 1-way ANOVA and post hoc Dunnett’s test for multiple comparison. For nonnormally distributed data, statistical analysis was performed using the Mann-Whitney U test with Dunnett’s multiple comparison. Value and error bars are represented as mean ± SD. *P* values less than 0.05 were considered statistically significant. Illustrations were made using Biorender.com. Image and Western blot analysis was performed using ImageJ software (44).

### Data availability.

The reconstructed map of PREPL Arg243C has been deposited to the Electron Microscopy Data Bank (EMDB) with the accession number EMD-19117. The atomic model is deposited in the Protein Data Bank (PDB) with accession number PDB-8RFB. The raw cryo-EM images will be deposited in the EMPIAR database.

## Author contribution

YM, KR, KB, AM, RV, SM, SV, and PA conducted the biochemical and cell biology experiments. AT, EC, AA, MJM, and MDP conducted the biophysical and structural experiments as well as in silico structural experiments. IL provided MAPPIT tools and performed the initial MAPPIT screen. JAL, CJS, RJS, RAM, CGL, DJP, and AR identified CMS22 patients and described patient characteristics. Experiments were designed by JWMC, MDP, MJM, YM, KR, and AT. Manuscript was written by YM, AT, KR, MJM, AR, MDP, and JWMC.

## Supplementary Material

Supplemental data

Unedited blot and gel images

Supporting data values

## Figures and Tables

**Table 1:**
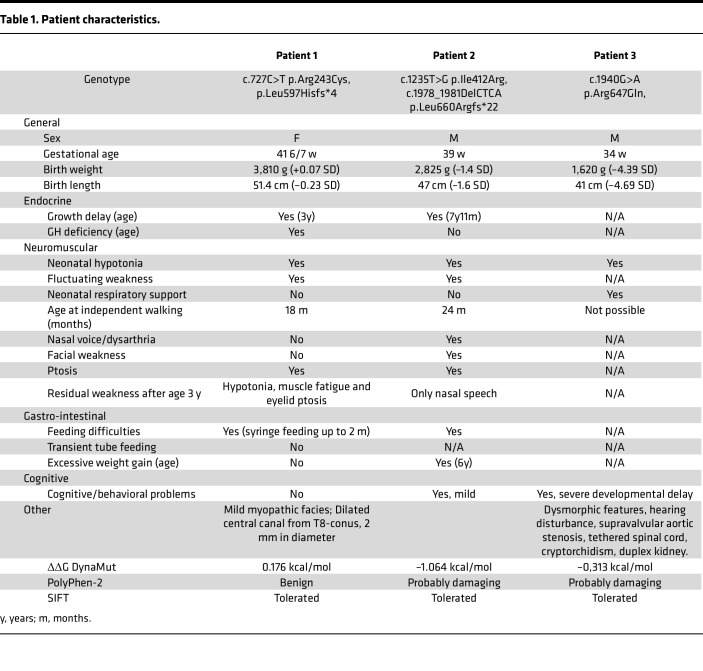
Patient characteristics.

## Version 1 07/30/2024

In-Press Preview

## Version 2 09/10/2024

Electronic publication
