# Supplementary material for: Missense variants in CMS22 patients reveal that PREPL has both enzymatic and nonenzymatic functions
Source: JCI Insight. 2024 Sep 10;9(17):e179276. doi: 10.1172/jci.insight.179276 (PMC11385081; doi:10.1172/jci.insight.179276)
Supplement: Supplemental data [file jciinsight-9-179276-s072.pdf]

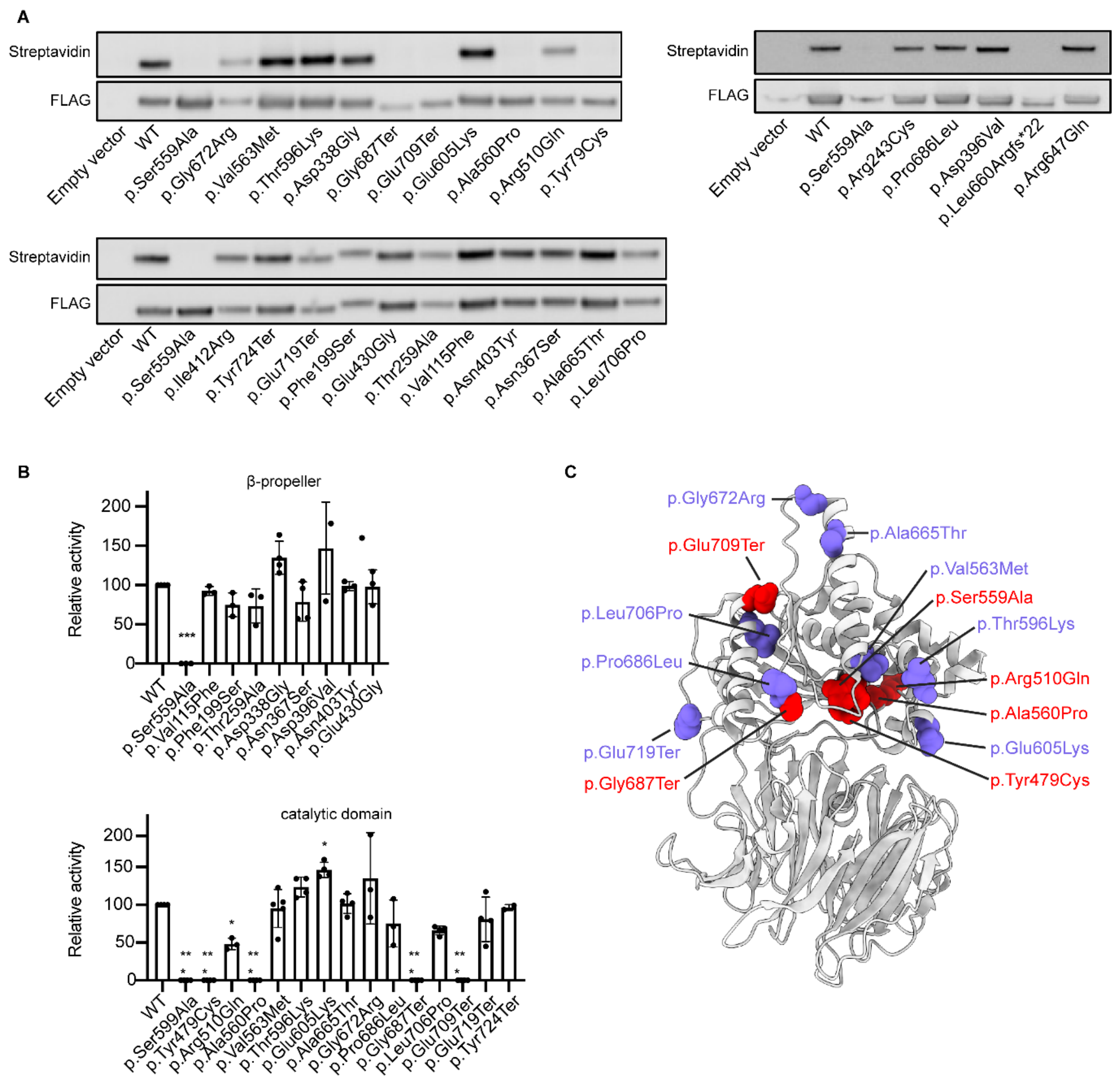

Figure S1: FP-biotin activity-based probe assay on PREPL variants obtained from the ClinVar database

(**A**) FP-biotin – streptavidin blots depicting relative FP-biotin binding in WT and PREPL variants. **(B)** Quantitative FP-biotin binding in WT and PREPL variants normalized to total PREPL abundance. (n=3) **(C)** Relative location within the protein structure of the variants in the catalytic domain. Statistical analysis was performed using one-way ANOVA. Significance levels are shown as * $p \leq 0.05$ ** $p \leq 0.01$, *** $p \leq 0.001$ and **** $p \leq 0.0001$

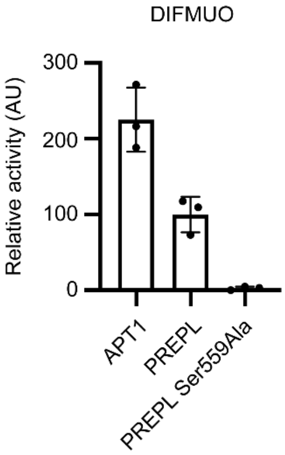

Figure S2: PREPL can cleave DIFMUO at 50% efficiency compared to APT1 DIFMUO substrate cleavage assay was performed for APT1, PREPL and PREPL p.Ser559Ala variant to validate substrate cleavage capacity of PREPL. PREPL has 50% cleavage efficiency compared to APT1 (n=3).

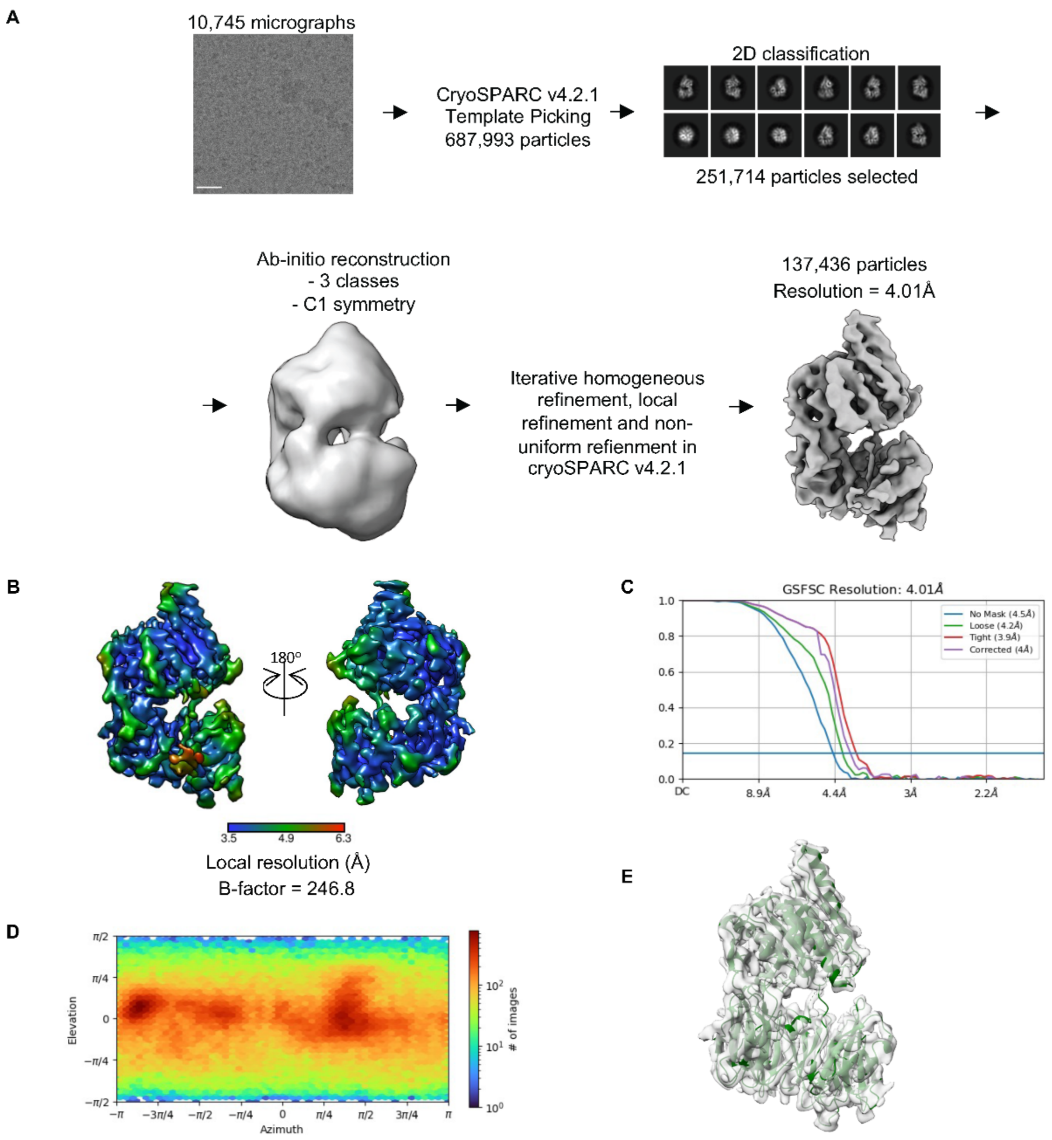

Figure S3: Cryo-EM analysis of PREPL p.Arg243Cys

(A) Flow chart of data processing. **(B)** Final 3D reconstruction of PREPL Arg243Cys, colored according to the local resolution. **(C)** Corrected Gold-standard Fourier shell correlation curves for the 3D electron microscopy reconstruction. **(D)** Angular distribution of PREPL Arg243Cys particles included in the final reconstruction. **(E)** PREPL Arg243Cys model fitted into the map.

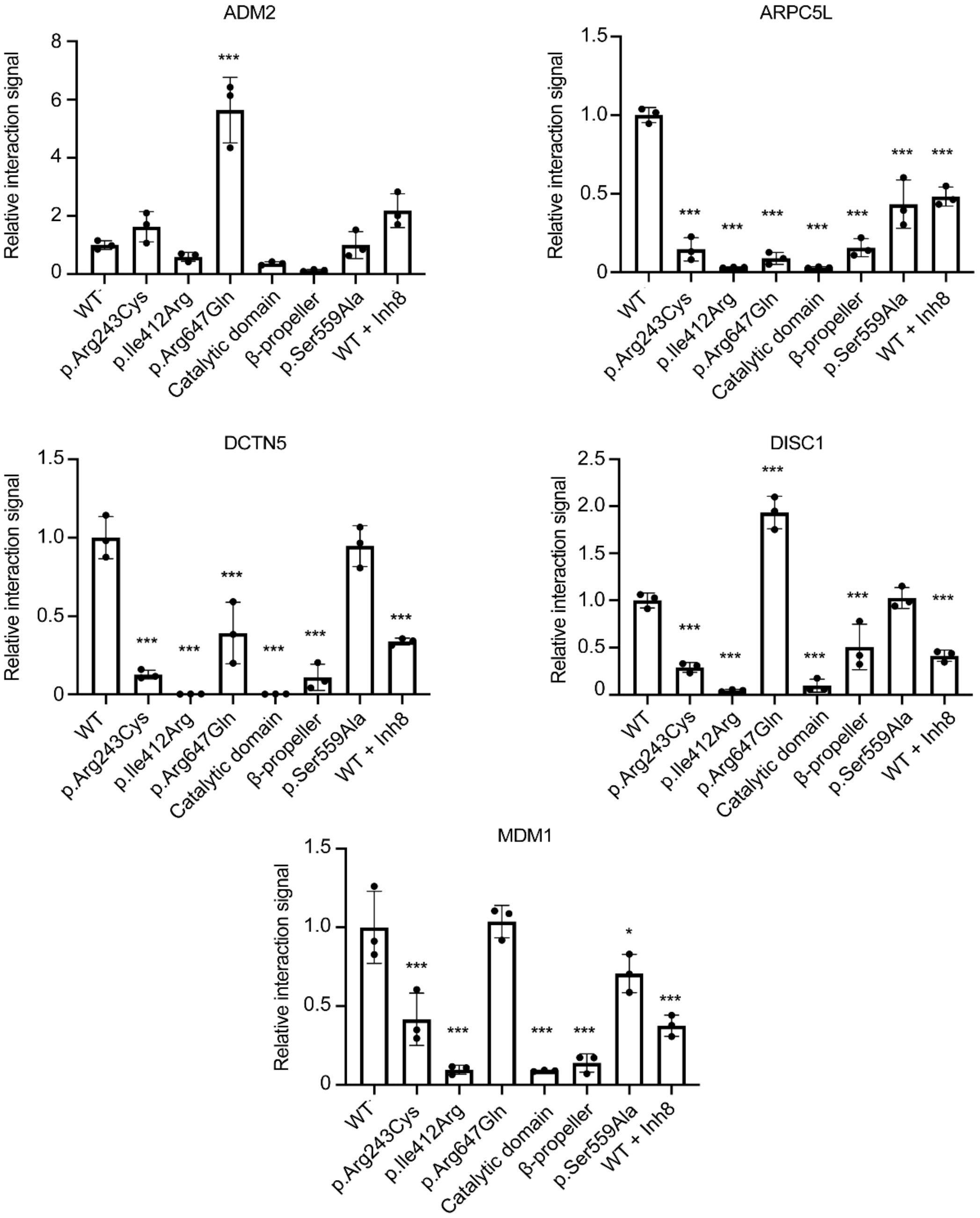
ADM2
ARPC5L
DCTN5
DISC1
MDM1
Relative interaction signal
WT
p.Arg243Cys
p.Ile412Arg
p.Arg647Gln
Catalytic domain
β-propeller
p.Ser559Ala
WT + Inh8

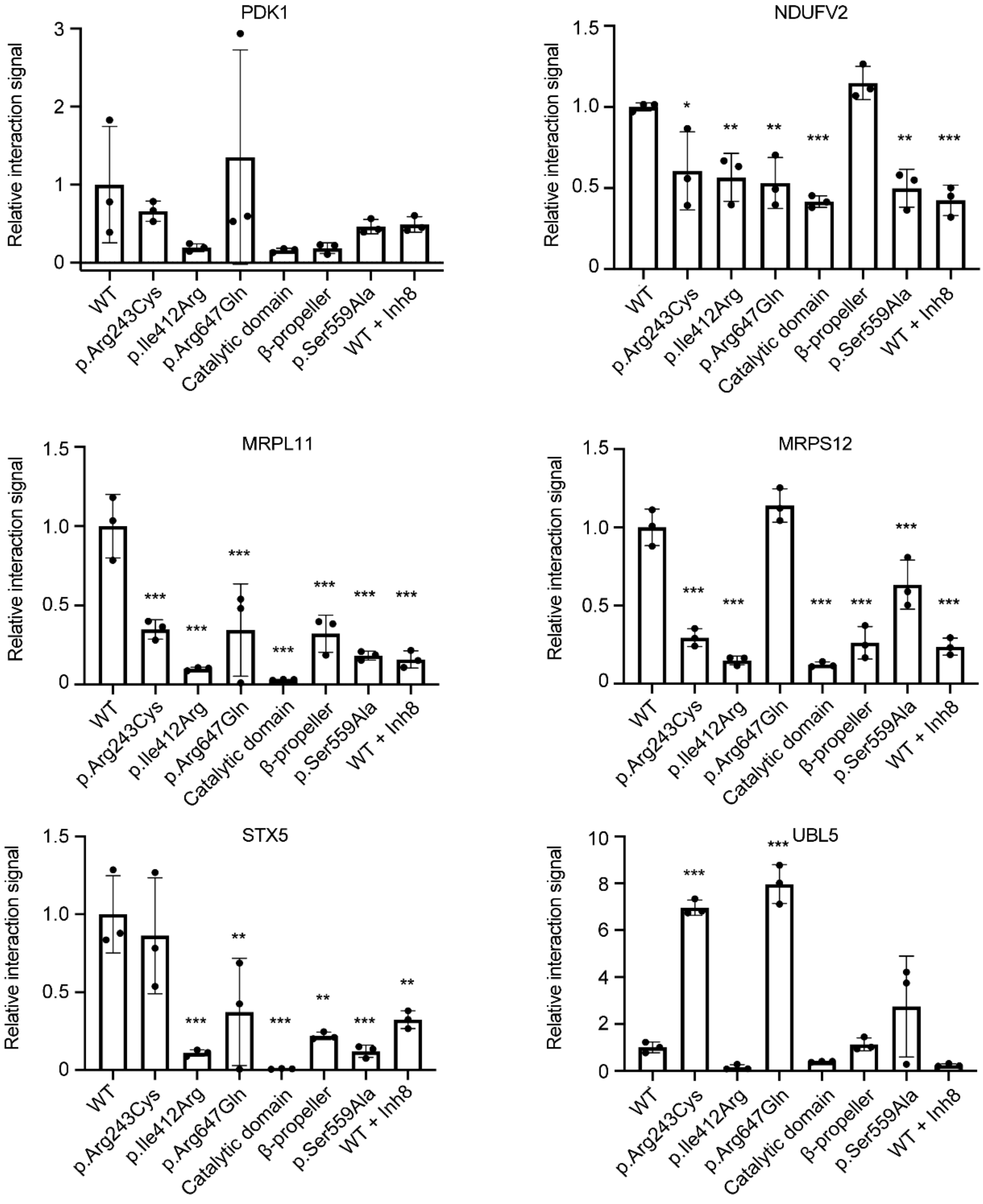

Figure S4: Detailed results of MAPPIT analysis of protein-protein interaction scores

MAPPIT protein-protein interaction scored depicted for each of the 11 previously identified interactor partners with eight PREPL variants (n=3). Statistical analysis was performed using one-way ANOVA. Significance levels are shown as * $p \leq 0.05$ ** $p \leq 0.01$, *** $p \leq 0.001$ and **** $p \leq 0.0001$.

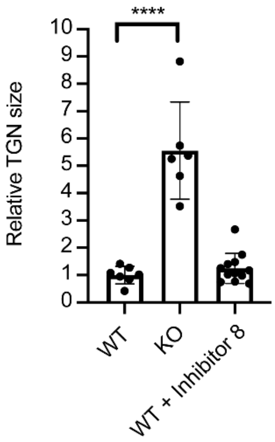

Figure S5: Evaluation of Trans-Golgi network size

Relative TGN size measured in WT, KO and WT treated with inhibitor 8 HEK293T cells (n=6-30). Addition of cell-permeable PREPL inhibitor 8 on WT HEK293T cells does not change the relative TNG size compared to non-treated WT HEK293T cells. Statistical analysis was performed by ANOVA. Significance levels are shown as * $p \leq 0.05$ ** $p \leq 0.01$, *** $p \leq 0.001$ and **** $p \leq 0.0001$

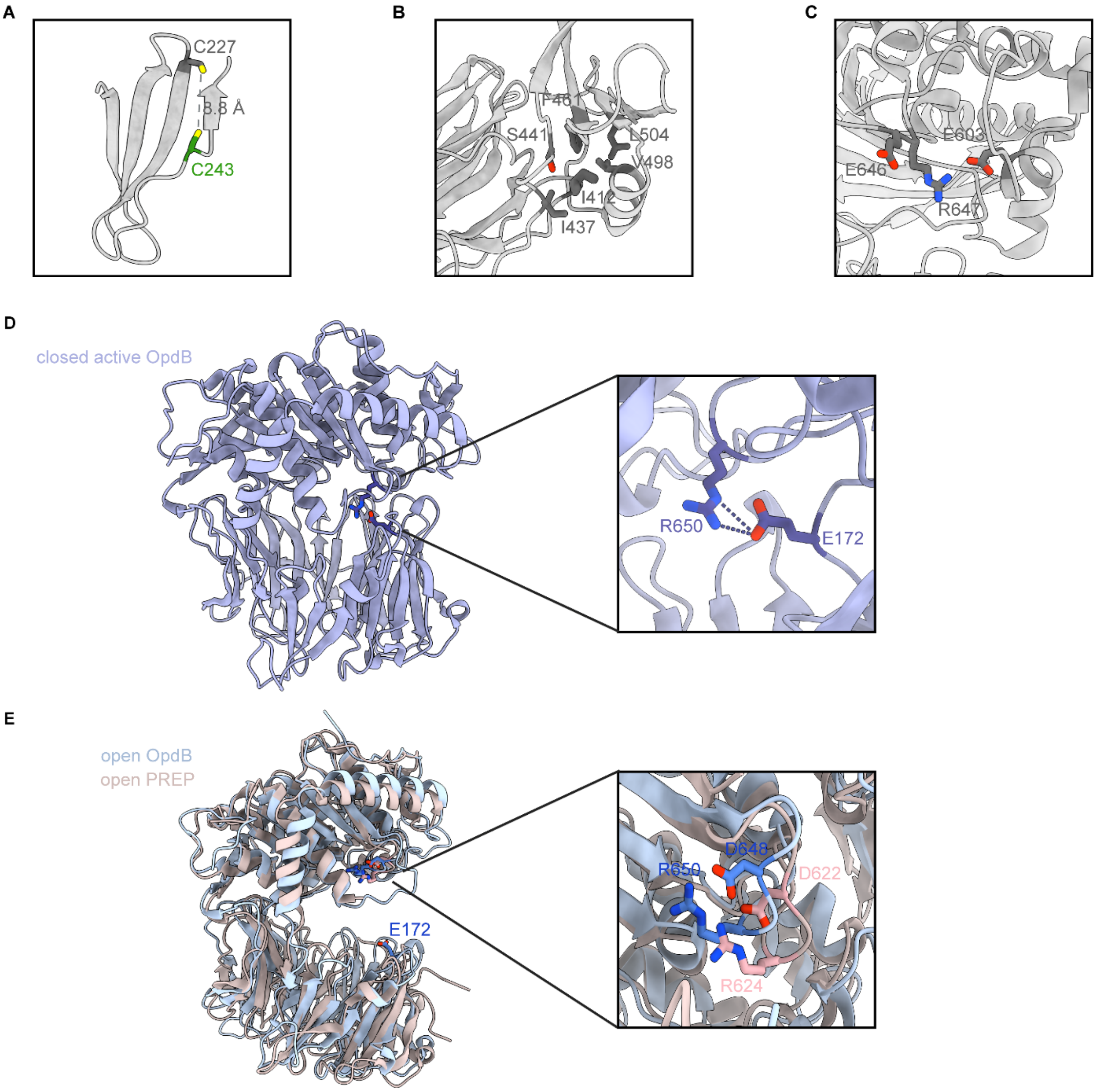

Figure S6: The effect of CMS22 mutations on protein structure

(A) The CMS22 Cys243 PREPL mutation and the WT Cys227 on the third blade of the b propeller, found within 9 Å distance. Despite the proximity of Cys227, the 9 Å distance between the two cysteine residues and the reducing environment of the cytoplasm makes the formation of a disulfide bond highly unlikely **(B)** The hydrophobic cluster formed on PREPL near Ile412 by Val498, Leu504, Ser441, Ile437, and Phe461. **(C)** Arg647 located near the catalytic domain residues Glu603 and Glu6466, implying a potential interaction between these residues. **(D)** The closed, active conformation of OpdB (PDB 4BP9), stabilized by the salt bridge formed between the catalytic domain Arg650 (Arg647 in PREPL) and the b-propeller residue Glu172 (Glu180 in PREPL based on structural superposition). **(E)** Superposition of the open conformations of OpdB (PDB 4BP8) and PREP (PDB 3IUL), highlighting the interactions formed by Arg650 in OpdB and Arg624 in PREP, the equivalent to PREPL Arg647 residues. In OpdB Glu172 (Glu180 in PREPL) is within a 16 Å distance from Arg650, rendering their interaction highly

unlikely. In this case, Arg650 interacts with Asp648 in OpdB. The same is observed in the closed conformation of PREP where Arg624 interacts with Asp622 of the catalytic domain.

Table S1. CryoEM map and atomic model refinement

**Access code**	
PDB	8RFB
EMDB	EMD-19117
**Data collection and processing**	
Microscope	TFS Titan Krios G4
Detector	Falcon IV
Recording mode	electron-counting mode (EER)
Magnification	250,000x
Voltage (kV)	300
Total dose ($e^-/Å^2$)	60
Nominal under focus range (µm)	1.5 - 2.5
Pixel size (Å)	0.3084
Movie micrograph exposure time (s)	1
Number of movie micrographs	10,745
Number of molecular projection images in map	251,714
Symmetry	C1
Map resolution (Å)	4.01
Map sharpening B-factor	246.8
**Model refinement and validation**	
Residues	596
Amino-acids	596
RMSD Bonds (4σ)	0.016
RMSD Angles (4σ)	1.579
**Ramachandran**	
Outliers (%)	0.00
Allowed (%)	1.86
Favored (%)	98.14
Rotamer outliers (%)	1.15
Clash score	1.82
Molprobity score	0.99
EMRinger score	2.39

Table S2. CRISPR-Cas9 oligos

Cell line	Forward	Reverse
***PREPL* KO guide RNA**	CACCGTGATACAATCAATGA AGGGC	AAACGCCCTTCATTGATTGTATCAC
**S559A mutant guide RNA**	CACCGTGACTGCTTTCAGTGC TGGA	AAACTCCAGCACTGAAAGCAGTCAC
**S559A mutant ssODN repair template**	CTCAATGGCCTTGCTGATTTAGAGGCTTGCATTAAGACGCTTCATGGCCAAGGCT TTTCTCAGCCAAGTCTAACAACCCTGACTGCTTTCGCAGCTGGAGGTGTGCTTGC AGGAGCATTGTGTAATTCTAATCCAGAGCTGGTGAGAGCGGTGACTTTGGAGGT GAGTACGCTCTGTCTCTACTGTTTATAG	
