# Supplementary material for: Missense variants in CMS22 patients reveal that PREPL has both enzymatic and nonenzymatic functions
Source: JCI Insight. 2024 Sep 10;9(17):e179276. doi: 10.1172/jci.insight.179276 (PMC11385081; doi:10.1172/jci.insight.179276)

Figure 1B

Streptavidin-HRP

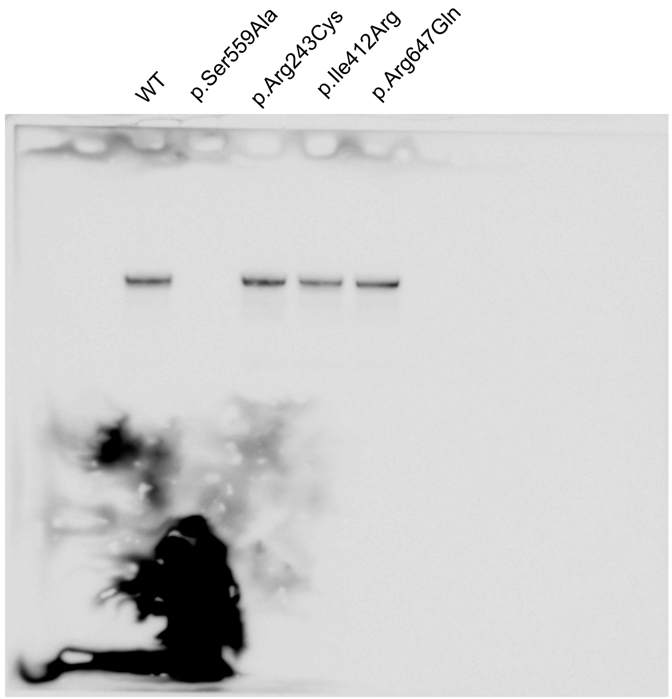

Flag M2

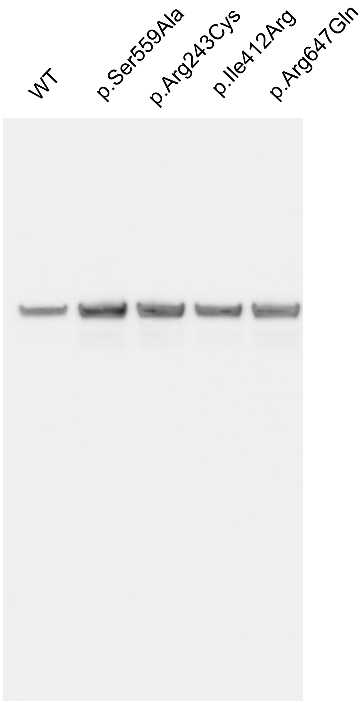

Figure 2E

Radioactive signal

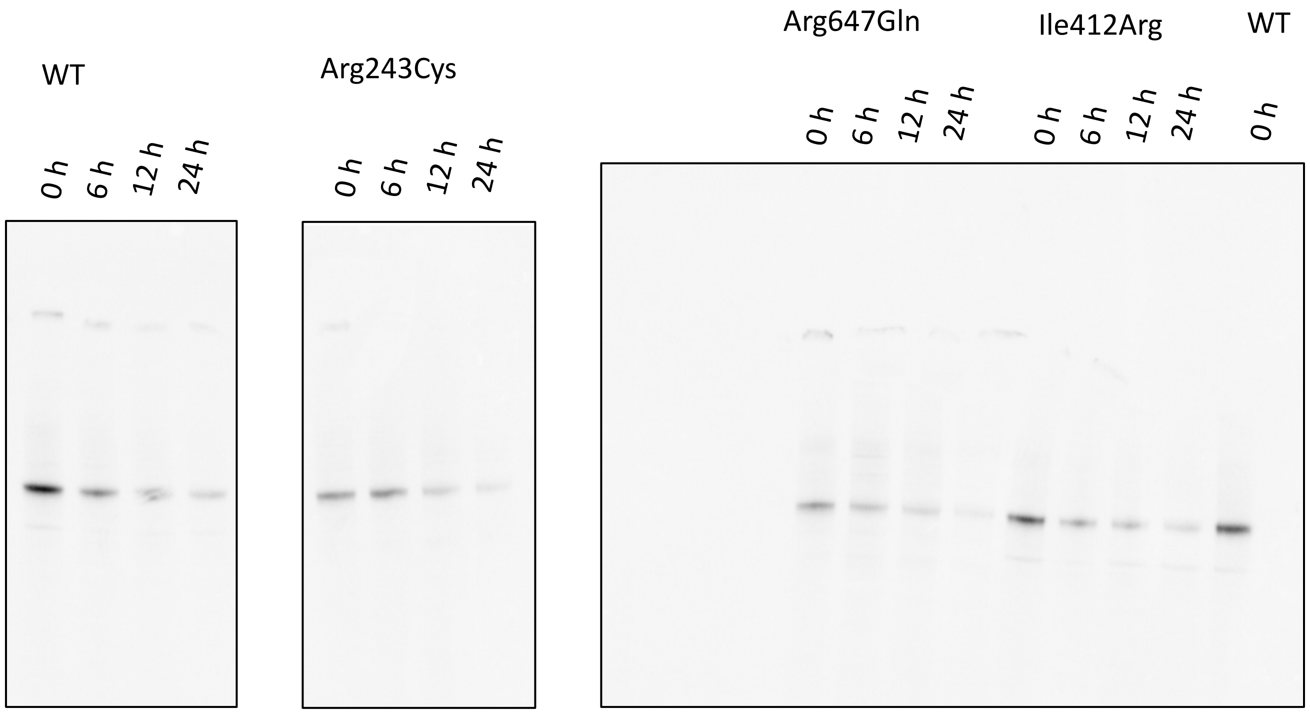

Figure 4A

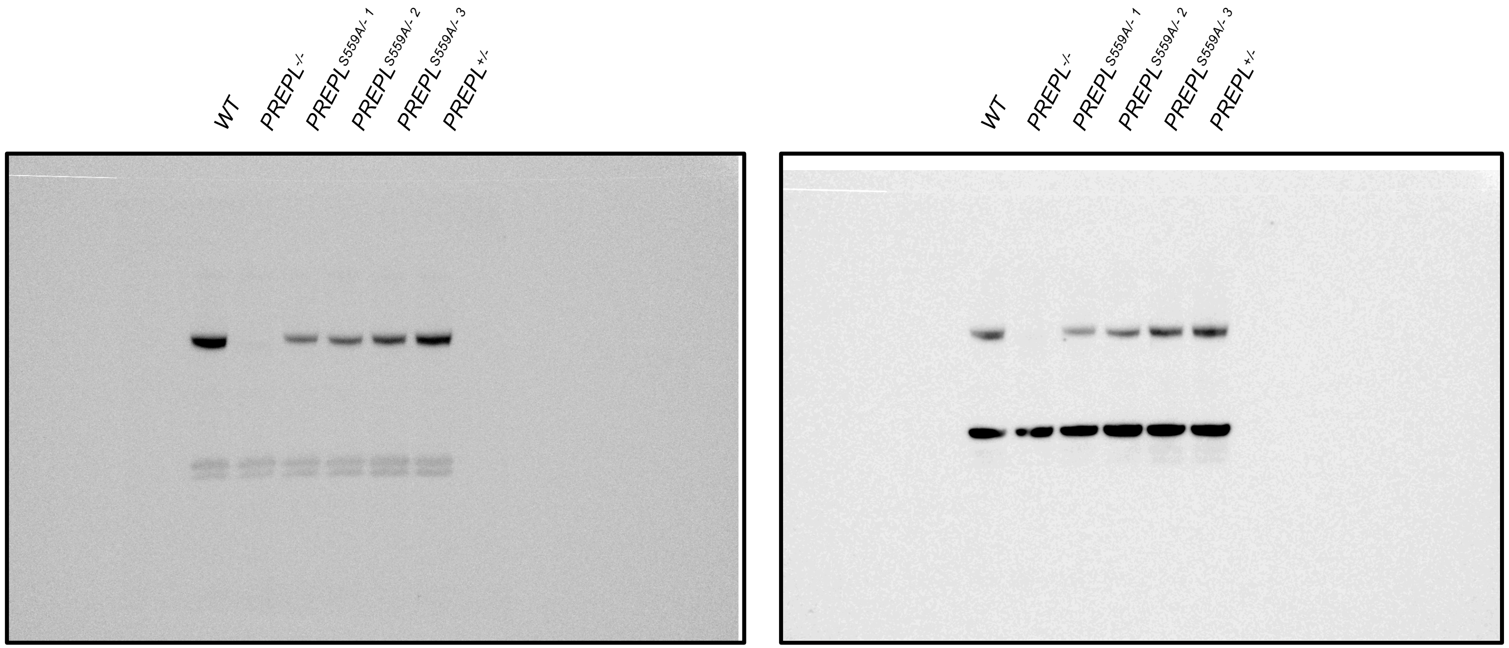
WT
PREPL$^{-/-}$
PREPL$^{S559A/- 1}$
PREPL$^{S559A/- 2}$
PREPL$^{S559A/- 3}$
PREPL$^{+/-}$
WT
PREPL$^{-/-}$
PREPL$^{S559A/- 1}$
PREPL$^{S559A/- 2}$
PREPL$^{S559A/- 3}$
PREPL$^{+/-}$

Figure 4B

FP-TAMRA fluorescent probe

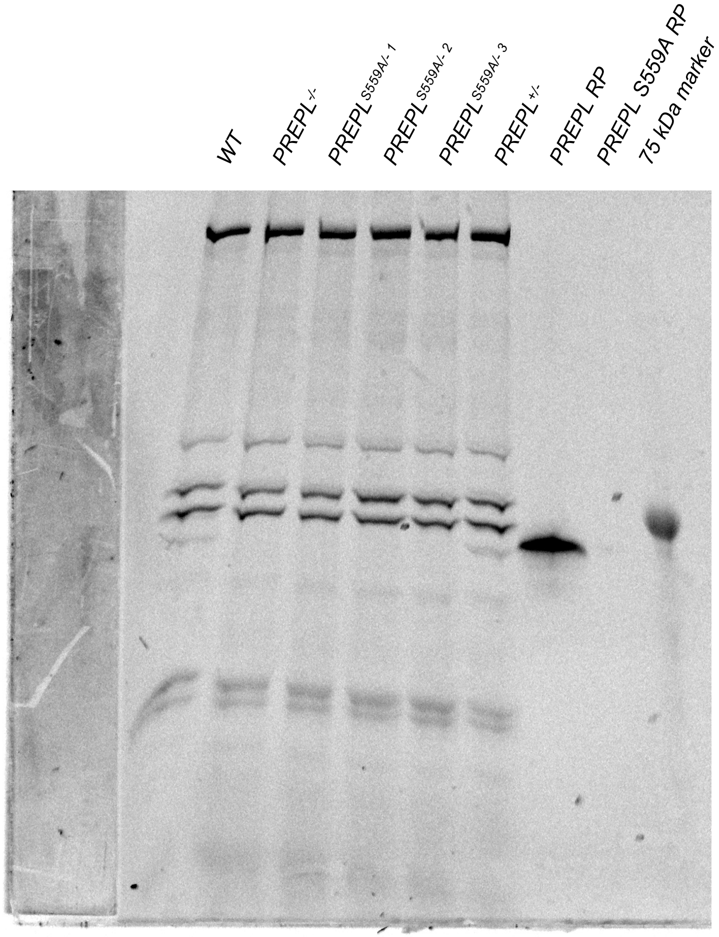

Figure S1A top

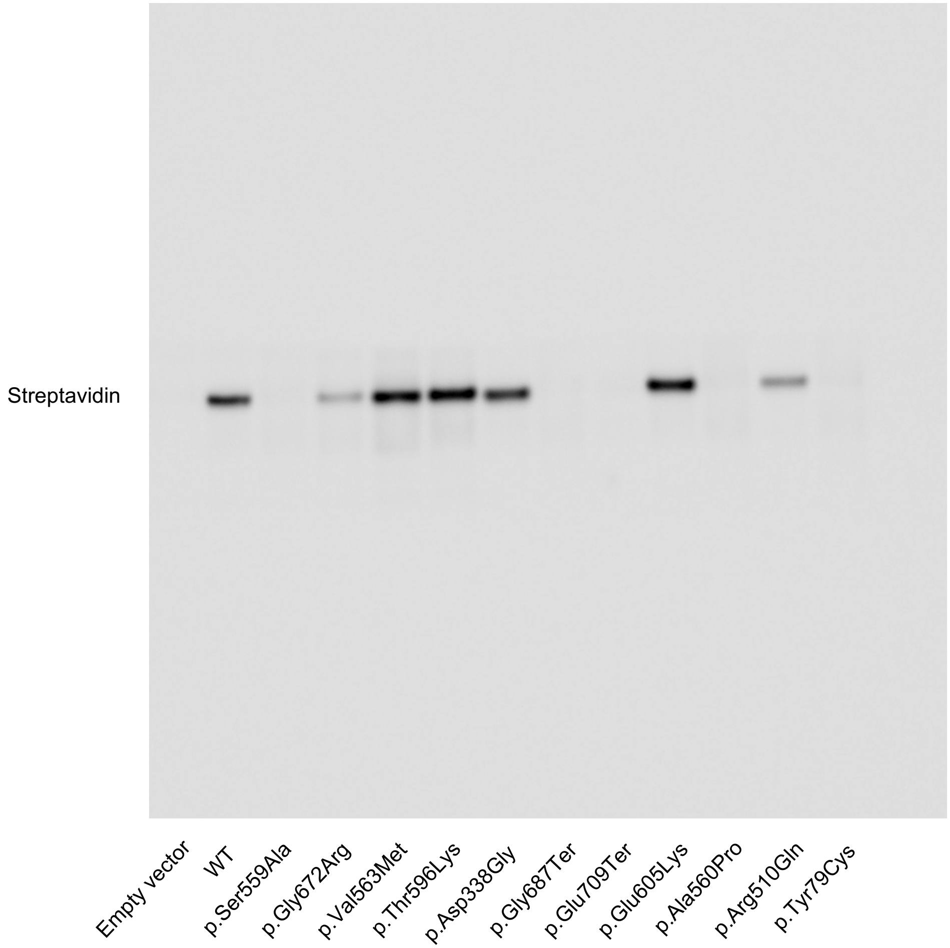

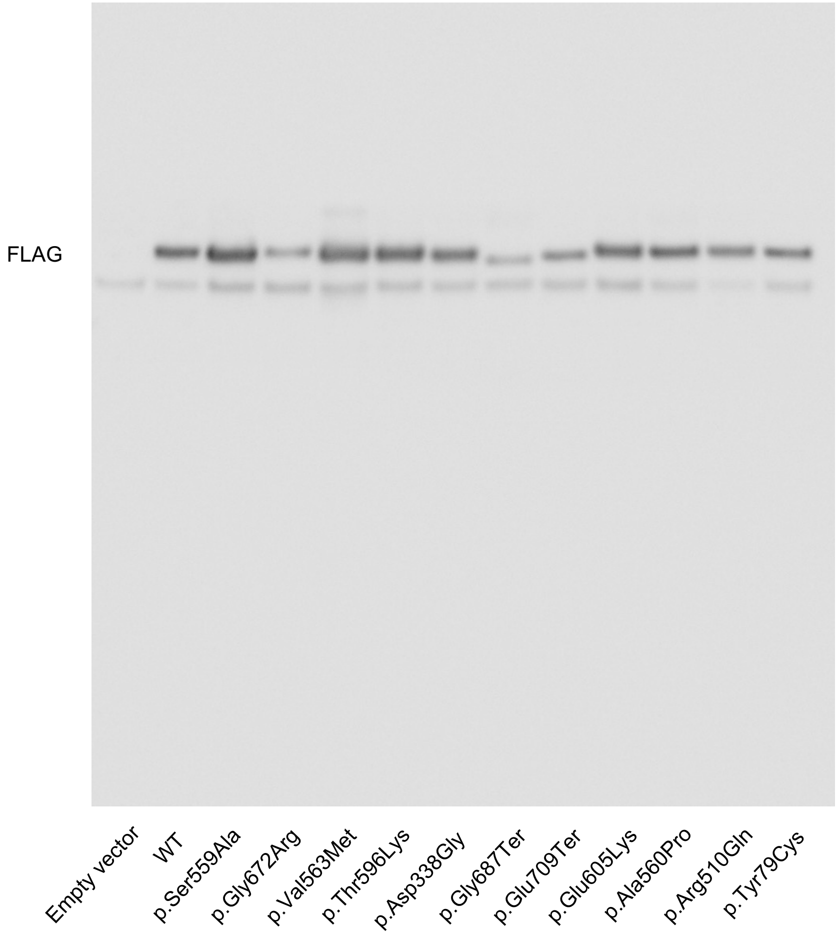

Figure S1A middle

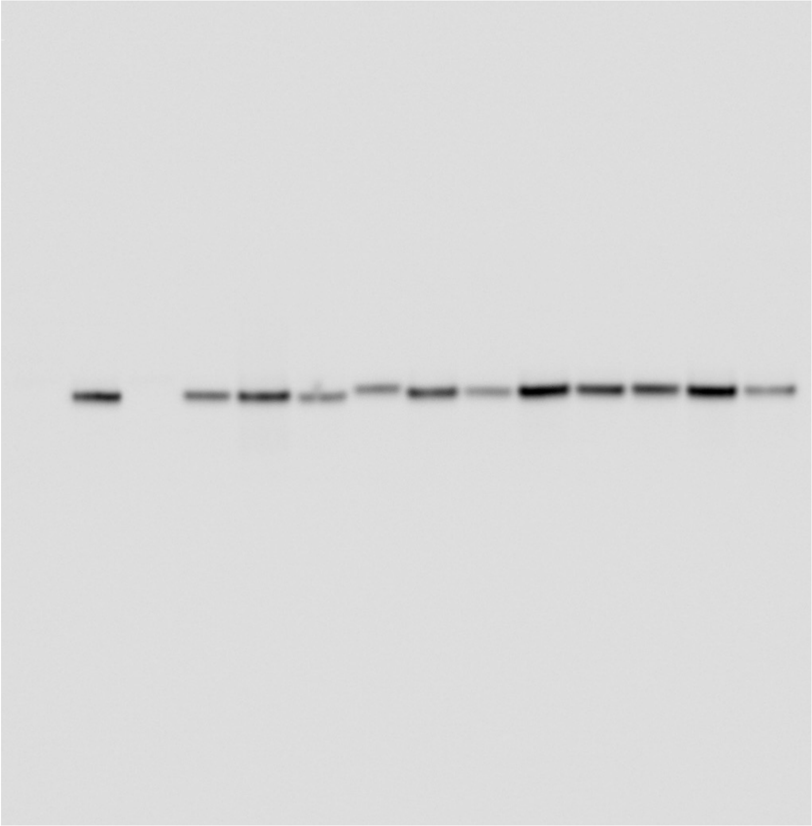

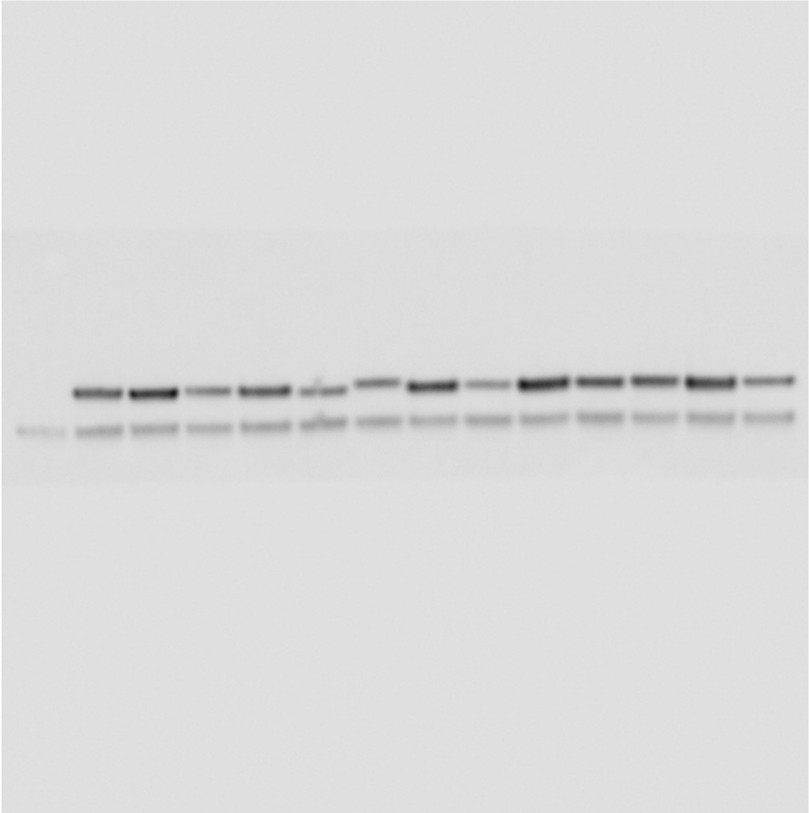

Figure S1A bottom

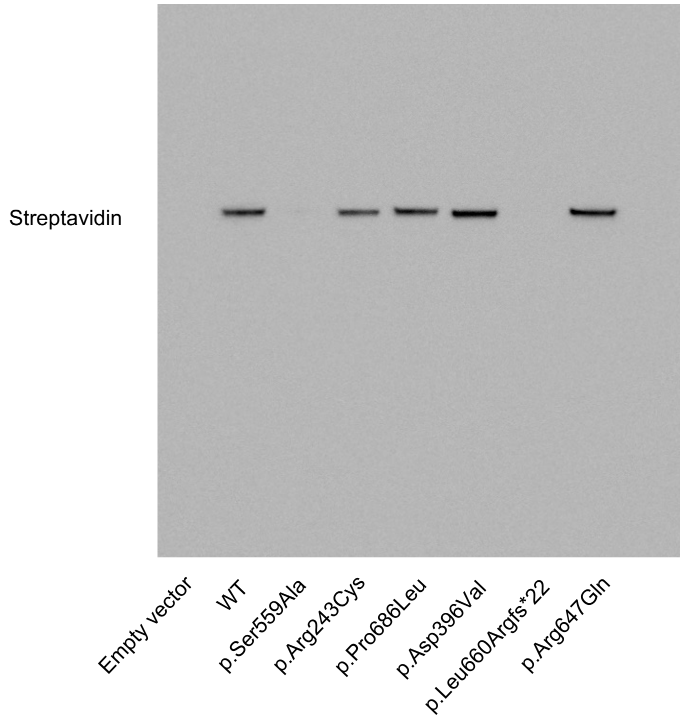

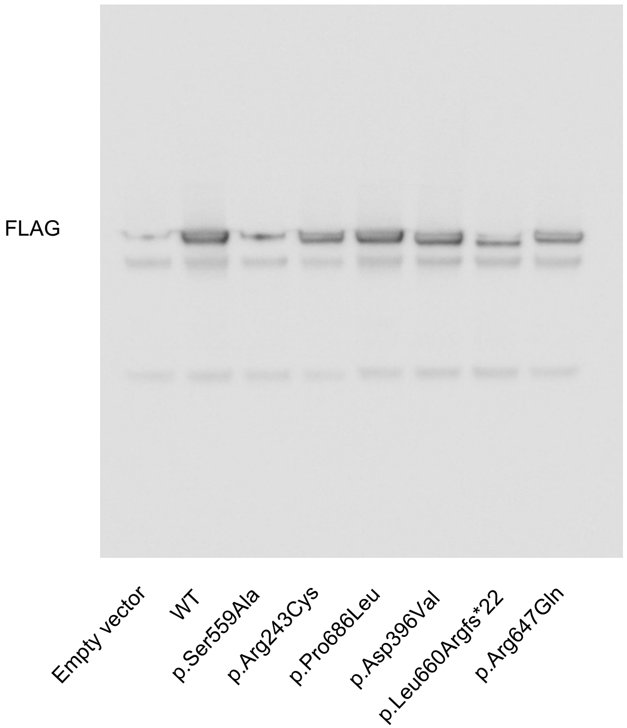

Supplement: Unedited blot and gel images [file jciinsight-9-179276-s073.pdf]
